# Supplementary material for: Acute and post-acute respiratory complications of SARS-CoV-2 infection: population-based cohort study in South Korea and Japan
Source: Nat Commun. 2024 May 27;15:4499. doi: 10.1038/s41467-024-48825-w (PMC11130304; doi:10.1038/s41467-024-48825-w)
Supplement: Supplementary file 1 — Supplementary Information [file 41467_2024_48825_MOESM1_ESM.pdf]

Supplementary Material

Original Article

Acute and post-acute respiratory complications of SARS-CoV-2: population-based cohort study in South Korea and Japan

Running head: **Adverse respiratory outcomes in post-acute COVID-19 condition**

Yujin Choi,[1,2‖] Hyeon Jin Kim,[1,2‖] Jaeyu Park,[1,2‖] Myeongcheol Lee,[1,3] Sunyoung Kim,[4] Ai Koyanagi,[5] Lee Smith,[6] Min Seo Kim,[7] Masoud Rahmati,[8,9,10] Hayeon Lee,[1*] Jiseung Kang,[11,12*] Dong Keon Yon[1,3,13*]

‖ These authors contributed equally as co-first authors.

* These authors contributed equally as corresponding authors

*Corresponding authors

Hayeon Lee, Ph.D.

Center for Digital Health, Medical Science research Institute, Kyung Hee University College of Medicine, 23 Kyungheedae-ro, Dongdaemun-gu, Seoul, 02447, South Korea
Email: wwhy28@khu.ac.kr

Jiseung Kang, Ph.D.

Department of Anesthesia, Critical Care and Pain Medicine, Massachusetts General Hospital, 149 13th Street, Room 4140, Charlestown, Boston, MA, USA

E-mail: wltmd1006@gmail.com

Dong Keon Yon, MD, PhD, FACAAI, FAAAAI (Lead contact)
Department of Pediatrics, Kyung Hee University College of Medicine, 23 Kyungheedae-ro, Dongdaemun-gu, Seoul, 02447, South Korea
Phone: +82-2-6935-2476
Fax: +82-504-478-0201
Email: yonkkang@gmail.com

Contents of Supplementary appendix

**Figure S1**	Study flow of cohorts
**Table S1**	Baseline characteristics for the full unmatched cohorts of South Korea (main cohort)
**Table S2**	Baseline characteristics for the full unmatched cohorts of Japan (replication cohort)
**Table S3**	Baseline characteristics for 1:3 propensity score–matched cohort (COVID-19 vs. general population) in Japan (replication)
**Table S4**	Baseline characteristics for 1:1 propensity score-matched cohort (COVID-19 vs. influenza) in South Korea (main)
**Table S5**	Baseline characteristics for 1:1 propensity score-matched cohort (COVID-19 vs. influenza) in Japan (replication)
**Table S6**	Hazard ratio (95% CI) for the **post-acute respiratory sequelae** or **acute respiratory complications** after SARS-CoV-2 infection in the propensity score-matched cohorts (COVID-19 vs. influenza) of South Korea (main) and Japan (replication)
**Table S7**	Baseline characteristics for the overlap-weighted cohort in South Korea (main)
**Table S8**	Baseline characteristics for the overlap-weighted cohort in Japan (replication)
**Table S9**	Overlap-weighted HR (95% CI) for the **post-acute respiratory sequelae** or **acute respiratory complications** subtypes following COVID-19 diagnosis of patients in binational cohorts (COVID-19 vs. general population) in South Korea (main) and Japan (replication)
**Table S10**	HR (95% CI) for the **post-acute respiratory sequelae** or **acute respiratory complications** subtypes after SARS-CoV-2 infection in the propensity score-matched cohorts (COVID-19 vs. influenza) in South Korea (main) and Japan (replication)
**Table S11**	Marginal predicted prevalence (percent; 95% CI) of general population *vs*. patients with COVID-19 in South Korea
**Table S12**	Marginal predicted prevalence (percent; 95% CI) of general population *vs*. patients with COVID-19 in Japan
**Table S13**	Propensity-score-matched subgroup analysis (COVID-19 vs. general population) of HR (95% CI) of **post-acute respiratory sequelae** or **acute respiratory complications** following COVID-19 diagnosis stratified by COVID-19 vaccination in South Korea

**Table S14**	Time attenuation effect analysis (COVID-19 vs. influenza) of HR (95% CI) for the risk of the **post-acute respiratory sequelae** after SARS-CoV-2 infection in South Korea (main cohort) and Japan (replication cohort)
**Table S15**	Baseline characteristics for 1:5 propensity score–matched cohort (positive for COVID-19 vs. negative for COVID-19 after the PCR test) in South Korea
**Table S16**	Stratification analysis for the risk of **post-acute respiratory sequelae** following COVID-19 in the propensity score matching cohorts (COVID-19 vs. general population) of South Korea (main)
**Table S17**	Stratification analysis for the risk of **post-acute respiratory sequelae** following COVID-19 in the propensity score matching cohorts of (COVID-19 vs. influenza) South Korea (main)
**Table S18**	Stratification analysis for the risk of **acute respiratory complications** following COVID-19 in the propensity score matching cohorts (COVID-19 vs. general population) of South Korea (main)
**Table S19**	Stratification analysis for the risk of **acute respiratory complications** following COVID-19 in the propensity score matching cohorts (COVID-19 vs. influenza) of South Korea (main)
**Table S20**	Stratification analysis for the risk of **post-acute respiratory sequelae** following COVID-19 in the propensity score matching cohorts (COVID-19 vs. general population) of Japan (replication)
**Table S21**	Stratification analysis for the risk of **post-acute respiratory sequelae** following COVID-19 in the propensity score matching cohorts (COVID-19 vs. influenza) of Japan (replication)
**Table S22**	Stratification analysis for the risk of **acute respiratory complications** following COVID-19 in the propensity score matching cohorts (COVID-19 vs. general population) of Japan (replication)
**Table S23**	Stratification analysis for the risk of **acute respiratory complications** following COVID-19 in the propensity score matching cohorts (COVID-19 vs. influenza) of Japan (replication)
**Table S24**	HR (95% CI) for the **post-acute respiratory sequelae** or **acute respiratory complications** subtypes after the PCR test in the propensity score-matched cohorts (COVID-19 vs. general population) in South Korea
**Table S25**	Definitions of diseases
**Table S26**	Statistical analyses and justification
**Table S27**	HR (95% CI) for the **post-acute respiratory sequelae** in **negative control analysis** using non-COVID-19 disease (tympanic membrane perforation) in the propensity score-matched main cohort (South Korea) and replication cohort (Japan)
**Supplementary Material**	Description of JMDC cohort

Figure S1. Study population in the main cohort (South Korea) and replication cohort (Japan)

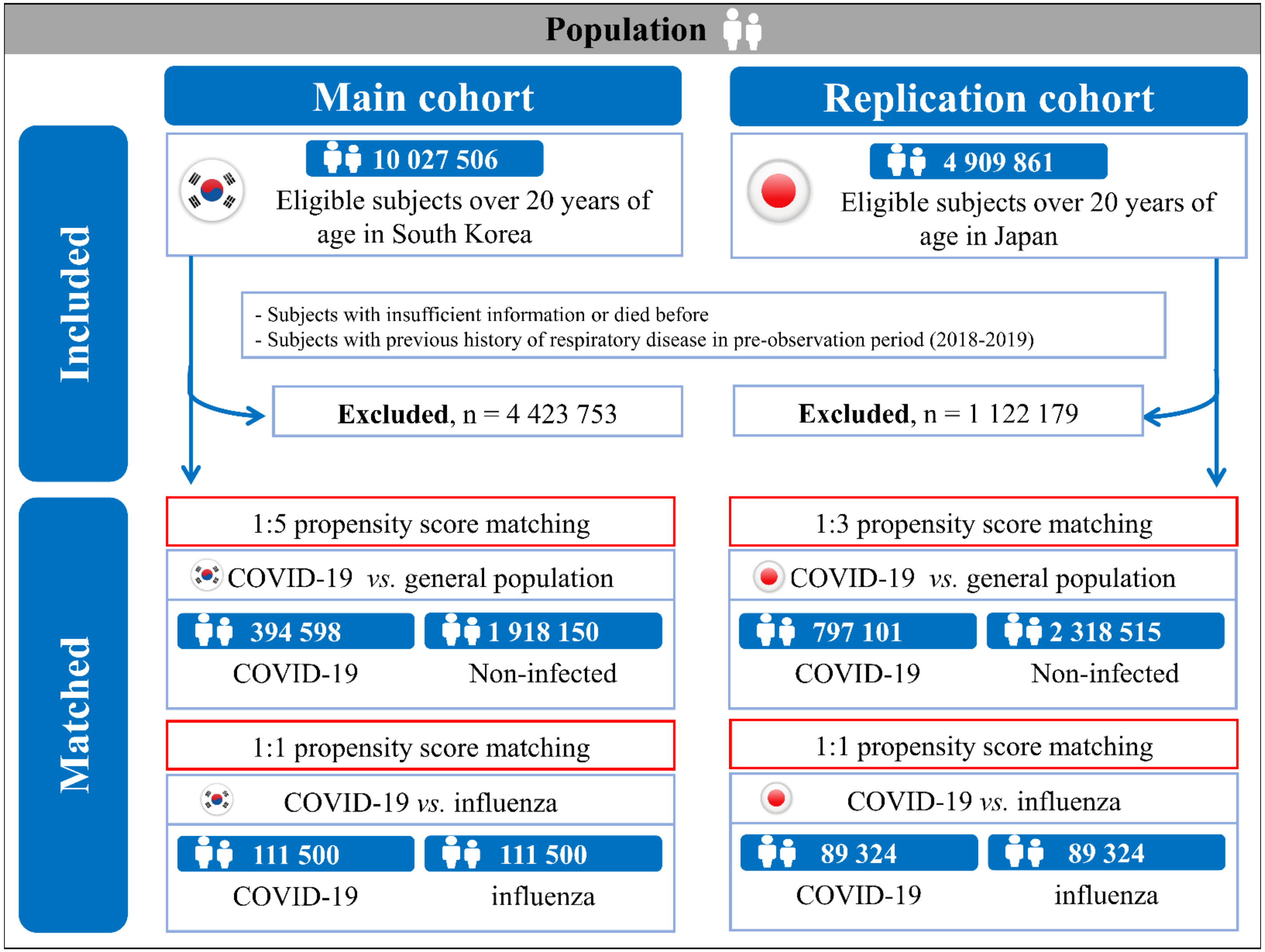
Population
Main cohort
Replication cohort
Included
10 027 506
Eligible subjects over 20 years of age in South Korea
4 909 861
Eligible subjects over 20 years of age in Japan
- Subjects with insufficient information or died before
- Subjects with previous history of respiratory disease in pre-observation period (2018-2019)
Excluded, n = 4 423 753
Excluded, n = 1 122 179
Matched
1:5 propensity score matching
COVID-19 vs. general population
394 598
COVID-19
1 918 150
Non-infected
1:3 propensity score matching
COVID-19 vs. general population
797 101
COVID-19
2 318 515
Non-infected
1:1 propensity score matching
COVID-19 vs. influenza
111 500
COVID-19
111 500
influenza
1:1 propensity score matching
COVID-19 vs. influenza
89 324
COVID-19
89 324
influenza

Figure S2. Study flow of cohorts

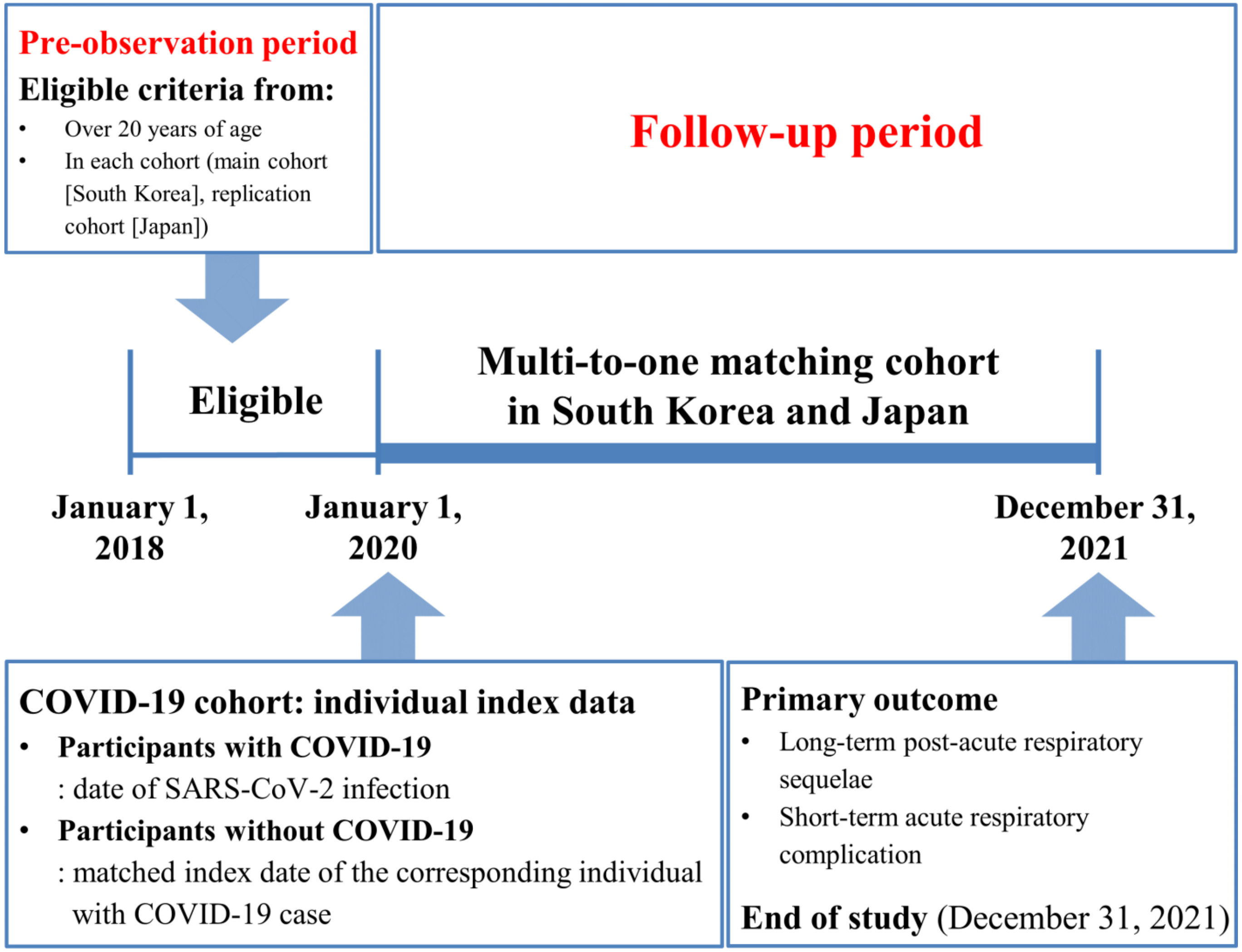

Table S1. Baseline characteristics for the full unmatched cohorts of South Korea (main cohort)

Characteristics	Main cohort
Total, n	10,027,506
Age, years, mean (SD)	48.4 (13.4)
Age, years, n (%)	
20 to 39	2,756,102 (27.5)
40 to 59	4,799,784 (47.9)
≥ 60	2,471,620 (24.7)
Sex, n (%)	
Male	5,026,885 (50.1)
Female	5,000,621 (49.9)
Region of residence, n (%)	
Rural	4,460,562 (44.5)
Urban	5,566,944 (55.5)

SD, standard deviation.

Table S2. Baseline characteristics for the full unmatched cohorts of Japan (replication cohort)

Characteristics	Replication cohort
Total, n	4,909,861
Age, years, mean (SD)	46.8 (11.9)
Age, years, n (%)	
20 to 39	1,362,496 (27.8)
40 to 59	2,801,078 (57.1)
≥ 60	746,287 (15.2)
Sex, n (%)	
Male	3,027,687 (61.7)
Female	1,882,174 (38.3)
Insurance status, n (%)	
Insured	4,291,062 (87.4)
Dependent	618,799 (12.6)

SD, standard deviation.

Table S3. Baseline characteristics for 1:3 propensity score–matched cohort (COVID-19 vs. general population) in Japan (replication)

**Characteristic**	COVID-19 *vs.* general population (n=3,115,606)		
	COVID-19 (n=797,101)	General population (n=2,318,505)	SMD*
**Mean age (SD),** ***y***	44 (11.88)	44 (12.03)	0.034
**Age,** ***n (%)***			<0.001
20–39 y	302,404 (37.94)	878,119 (37.87)	
40–59 y	411,351 (51.61)	1,199,717 (51.75)	
≥60 y	83,346 (10.46)	240,669 (10.38)	
**Sex,** ***n (%)***			0.005
Male	495,460 (62.16)	1,447,190 (62.42)	
Female	301,641 (37.84)	871,315 (37.58)	
**Insurance status,** ***n (%)***			0.001
Insured	718,811 (90.18)	2,091,308 (90.20)	
Dependent	78,290 (9.82)	227,197 (9.80)	
**Medical history,** ***n (%)***			
Cardiovascular disease	60,450 (7.58)	170,601 (7.36)	0.009
Chronic kidney disease	32292 (4.05)	90680 (3.91)	0.007

Medication use for diabetes	19,777 (2.48)	56,919 (2.45)	0.002
Medication use for hyperlipidemia	49,255 (6.18)	141,631 (6.11)	0.003
Medication use for hypertension	67,167 (8.43)	191,951 (8.28)	0.005
**Unmatching covariates, *n (%)*** †			
Charlson Comorbidity Index score			0.203
0	764,052 (95.85)	2,293,774 (98.93)	
1	10,954 (1.37)	8640 (0.37)	
≥2	22,095 (2.77)	16,091 (0.69)	
Body mass index			<0.001
Underweight (<18.5 kg/m^2)	427,244 (53.60)	1,263,598 (54.50)	
Normal (18.5-22.9 kg/m^2)	151,460 (19.00)	439,941 (18.98)	
Overweight (23.0-24.9 kg/m^2)	169,962 (21.32)	484,142 (20.88)	
Obese (≥25.0 kg/m^2)	47,210 (5.92)	127,534 (5.50)	
Unknown	1225 (0.15)	3290 (0.14)	
Blood pressure			0.038
SBP <140 mmHg and DBP <90 mmHg	691,676 (86.77)	1,986,000 (85.66)	
SBP ≥140mmHg or DBP ≥90 mmHg	46,474 (5.83)	149,954 (6.47)	
Unknown	58,951 (7.40)	182,551 (7.87)	

Fasting blood glucose			<0.001
<100 mg/dL	511,681 (64.19)	1,474,020 (63.58)	
≥100 mg/dL	160,434 (20.13)	474,726 (20.48)	
Unknown	124,986 (15.68)	369,759 (15.95)	
Serum total cholesterol			0.054
<200 mg/dL	373,377 (46.84)	1,040,983 (44.90)	
200 to 239 mg/dL	285,475 (35.81)	843,032 (36.36)	
≥240 mg/dL	123,823 (15.53)	379,819 (16.38)	
Unknown	14,426 (1.81)	54,671 (2.36)	
Glomerular filtration rate			0.037
<60 mL/min/1.73 m^2	4383 (0.55)	8174 (0.35)	
60 to 89 mL/min/1.73 m^2	72,932 (9.15)	215,112 (9.28)	
≥90 mL/min/1.73 m^2	433,528 (54.39)	1,237,792 (53.39)	
Unknown	286,258 (35.91)	857,427 (36.98)	
Smoking status			0.062
Non-smoker	181,033 (22.71)	554,190 (23.90)	
Smoker	586,552 (73.59)	1,677,800 (72.37)	
Unknown	29,516 (3.70)	86,515 (3.73)	

Alcohol consumption			0.040
Everyday	161,809 (20.30)	466,183 (20.11)	
Sometimes	264,583 (33.19)	762,501 (32.89)	
Rarely	311,632 (39.10)	919,772 (39.67)	
Unknown	59,077 (7.41)	170,049 (7.33)	
Aerobic physical activity			0.046
Insufficient	161,205 (20.22)	497,575 (21.46)	
Sufficient	562,300 (70.54)	1,605,294 (69.24)	
Unknown	73,596 (9.23)	215,636 (9.30)	
Strain of SARS-CoV-2			0.005
Original	335,571 (42.10)	981,871 (42.35)	
Delta	461,530 (57.90)	1,336,634 (57.65)	

DBP, diastolic blood pressure; SARS-CoV-2, severe acute respiratory syndrome coronavirus 2; SBP, systolic blood pressure; SD, standard deviation; SMD, standardized mean difference.

* An SMD <0.1 indicates no significant imbalance. All SMDs were <0.1 in the propensity score–matched cohorts.

† Unmatched covariates were included as adjustment factors in statistical analyses.

Table S4. Baseline characteristics for 1:1 propensity score-matched cohort (COVID-19 vs. influenza) in South Korea (main)

**Characteristic**	COVID-19 *vs.* influenza (n=223,000)		
	COVID-19 (n=111,500)	Influenza (n=111,500)	SMD*
**Mean age (SD), *y***	45.3 (15.3)	45.0 (12.7)	0.017
**Age, *n (%)***			0.060
20–39 y	42,625 (38.2)	40,131 (36.0)	
40–59 y	50,713 (45.5)	53,295 (47.8)	
≥60 y	18,162 (16.3)	18,074 (16.2)	
**Sex, *n (%)***			0.046
Male	46,400 (41.6)	49,587 (44.5)	
Female	65,100 (58.4)	61,913 (55.5)	
**Region of residence, *n (%)***			0.005
Urban	49,431 (44.3)	49,160 (44.1)	
Rural	62,069 (55.7)	62,340 (55.9)	
**Medical history, *n (%)***			
Cardiovascular disease	4166 (3.7)	4258 (3.8)	0.004
Chronic kidney disease	2011 (1.8)	1991 (1.8)	0.001
Medication use for diabetes	17,218 (15.4)	16,665 (15.0)	0.014
Medication use for hyperlipidemia	17,656 (15.8)	17,167 (15.4)	0.012
Medication use for hypertension	7408 (6.6)	6640 (6.0)	0.028
**Unmatching covariates, *n (%)***†			
Charlson Comorbidity Index score			0.103
0	99,673 (89.4)	103,133 (92.5)	

1	7867 (7.1)	5296 (4.8)	
≥2	3960 (3.6)	3071 (2.8)	
Household income			0.078
Low (0th–39th percentile)	48,878 (43.8)	46,012 (41.3)	
Middle (40th –79th percentile)	45,339 (40.7)	45,119 (40.5)	
High (80th–100th percentile)	17,283 (15.5)	20,369 (18.3)	
Body mass index			1.180
Underweight (<18.5 kg/m^2)	2120 (1.9)	3997 (3.6)	
Normal (18.5-22.9 kg/m^2)	22,894 (20.5)	39,561 (35.5)	
Overweight (23.0-24.9 kg/m^2)	14,256 (12.8)	24,459 (21.9)	
Obese (≥25.0 kg/m^2)	25,159 (22.6)	43,474 (39.0)	
Unknown	47,071 (42.2)	9 (0.0081)	
Blood pressure			1.207
SBP <140 mmHg and DBP <90 mmHg	55,801 (50.1)	99,099 (88.9)	
SBP ≥140mmHg or DBP ≥90 mmHg	8400 (7.5)	12,237 (11.0)	
Unknown	47299 (42.4)	164 (0.15)	
Fasting blood glucose			1.229
<100 mg/dL	40,399 (36.2)	72,206 (64.8)	
≥100 mg/dL	23,797 (21.3)	39,131 (35.1)	
Unknown	47,304 (42.4)	163 (0.15)	
Serum total cholesterol			0.462
<200 mg/dL	17,459 (15.7)	29,991 (26.9)	
200 to 239 mg/dL	10485 (9.4)	18,760 (16.8)	
≥240 mg/dL	4859 (4.4)	7923 (7.1)	
Unknown	78,697 (70.6)	54,826 (49.2)	
Glomerular filtration rate			1.208

<60 mL/min/1.73 m^2	1689 (1.5)	2223 (2.0)	
60 to 89 mL/min/1.73 m^2	26,592 (23.9)	45,211 (40.6)	
≥90 mL/min/1.73 m^2	35,867 (32.2)	63,785 (57.2)	
Unknown	47,352 (42.5)	281 (0.25)	
Smoking status			1.155
Never	45,487 (40.8)	75,040 (67.3)	
Former	9450 (8.5)	15,936 (14.3)	
Current	9506 (8.5)	20,488 (18.4)	
Unknown	47,057 (42.2)	36 (0.032)	
Alcohol consumption			1.208
<1 day/week	38,511 (34.5)	68078 (61.1)	
1 to 2 days/week	17,887 (16.0)	31025 (27.8)	
3 to 4 days/week	6125 (5.5)	9620 (8.6)	
≥5 days/week	1922 (1.7)	2741 (2.5)	
Unknown	47,055 (42.2)	36 (0.032)	
Aerobic physical activity			1.228
Insufficient	33,898 (30.4)	58,183 (52.2)	
Sufficient	30,539 (27.4)	53,276 (47.8)	
Unknown	47,063 (42.2)	41 (0.037)	
Strain of SARS-CoV-2			<0.001
Original	34685 (31.1)	34,718 (31.1)	
Delta	76,815 (68.9)	76,782 (68.9)	

DBP, diastolic blood pressure; SARS-CoV-2, severe acute respiratory syndrome coronavirus 2;SBP, systolic blood pressure; SD, standard deviation; SMD, standardized mean difference.

* An SMD <0.1 indicates no significant imbalance. All SMDs were <0.1 in the propensity score–matched cohorts.

† Unmatched covariates were included as adjustment factors in statistical analyses.

Table S5. Baseline characteristics for 1:1 propensity score-matched cohort (COVID-19 vs. influenza) in Japan (replication)

**Characteristic**	COVID-19 *vs.* influenza (n=178,648)		
	COVID-19 (n=89,324)	Influenza (n=89,324)	SMD*
**Mean age (SD), *y***	44 (11.76)	44 (11.57)	0.018
**Age, *n (%)***			<0.001
20–39 y	31,256 (34.99)	31,210 (34.94)	
40–59 y	48,959 (54.81)	49,241 (55.13)	
≥60 y	9109 (10.20)	8873 (9.93)	
**Sex, *n (%)***			0.005
Male	55,617 (62.26)	55,396 (62.02)	
Female	33,707 (37.74)	33,928 (37.98)	
**Insurance status, *n (%)***			<0.001
Insured	80,141 (89.72)	80,141 (89.72)	
Dependent	9183 (10.28)	9183 (10.28)	
**Medical history, *n (%)***			
Cardiovascular disease	6235 (6.98)	6214 (6.96)	0.001
Chronic kidney disease	3031 (3.39)	3252 (3.64)	0.013
Medication use for diabetes	2422 (2.71)	2200 (2.46)	0.016
Medication use for hyperlipidemia	6159 (6.90)	5877 (6.58)	0.013
Medication use for hypertension	8016 (8.97)	7716 (8.64)	0.012

**Unmatching covariates, *n (%)*** †			
Charlson Comorbidity Index score			0.153
0	85,599 (95.83)	87,875 (98.38)	
1	1150 (1.29)	501 (0.56)	
≥2	2575 (2.88)	948 (1.06)	
Body mass index			0.007
Underweight (<18.5 kg/m^2)	47,532 (53.21)	47,767 (53.48)	
Normal (18.5-22.9 kg/m^2)	17,018 (19.05)	16,935 (18.96)	
Overweight (23.0-24.9 kg/m^2)	19,260 (21.56)	19,131 (21.42)	
Obese (≥25.0 kg/m^2)	5373 (6.02)	5366 (6.01)	
Unknown	125 (0.14)	141 (0.16)	
Blood pressure			<0.001
SBP <140 mmHg and DBP <90 mmHg	77,118 (86.34)	77,019 (86.22)	
SBP ≥140mmHg or DBP ≥90 mmHg	5456 (6.11)	5383 (6.03)	
Unknown	6750 (7.56)	6922 (7.75)	
Fasting blood glucose			0.028
<100 mg/dL	57,035 (63.85)	57,644 (64.53)	
≥100 mg/dL	18,237 (20.42)	18,284 (20.47)	
Unknown	14,052 (15.73)	13,396 (15.00)	
Serum total cholesterol			0.022
<200 mg/dL	41,317 (46.26)	39,896 (44.66)	

200 to 239 mg/dL	32,270 (36.13)	33,092 (37.05)	
≥240 mg/dL	14,211 (15.91)	14,672 (16.43)	
Unknown	1526 (1.71)	1664 (1.86)	
Glomerular filtration rate			0.073
<60 mL/min/1.73 m^2	501 (0.56)	296 (0.33)	
60 to 89 mL/min/1.73 m^2	8344 (9.34)	7987 (8.94)	
≥90 mL/min/1.73 m^2	48,818 (54.65)	46,549 (52.11)	
Unknown	31,661 (35.45)	34,492 (38.61)	
Smoking status			0.062
Non-smoker	20,354 (22.79)	21,458 (24.02)	
Smoker	65,734 (73.59)	64,706 (72.44)	
Unknown	3236 (3.62)	3160 (3.54)	
Alcohol consumption			0.049
Everyday	18,486 (20.70)	18,284 (20.47)	
Sometimes	29,295 (32.80)	28,687 (32.12)	
Rarely	35,096 (39.29)	35,716 (39.98)	
Unknown	6447 (7.22)	6637 (7.43)	
Aerobic physical activity			0.035
Insufficient	18,249 (20.43)	18,303 (20.49)	
Sufficient	62,979 (70.51)	62,858 (70.37)	
Unknown	8096 (9.06)	8163 (9.14)	
Strain of SARS-CoV-2			1.652
Original	36,886 (41.29)	88,835 (99.45)	
Delta	52,438 (58.71)	489 (0.55)	

DBP, diastolic blood pressure; SARS-CoV-2, severe acute respiratory syndrome coronavirus 2; SBP, systolic blood pressure; SD, standard deviation; SMD, standardized mean difference.

* An SMD <0.1 indicates no significant imbalance. All SMDs were <0.1 in the propensity score–matched cohorts.

† Unmatched covariates were included as adjustment factors in statistical analyses.

Table S6. Hazard ratio (95% CI) for the **post-acute respiratory sequelae** or **acute respiratory complications** after SARS-CoV-2 infection in the propensity score-matched cohorts (COVID-19 vs. influenza) of South Korea (main) and Japan (replication)

**Cohort**	**South Korea**			**Japan**		
	COVID-19 *vs.* influenza (n=223,000)			COVID-19 *vs.* influenza (n=169,924)		
		HR (95% CI)			HR (95% CI)	
	Events, *n (%)*	Model 1[*]	Model 2[†]	Events, *n (%)*	Model 3[*]	Model 4[‖]
**Post-acute respiratory sequelae**						
Comparators (general population or patients with influenza)	1081 (0.97)	1.0 (reference)	1.0 (reference)	905 (1.07)	1.0 (reference)	1.0 (reference)
Patients with COVID-19	1500 (1.35)	**1.55 (1.43-1.67)**	**1.66 (1.52-1.82)**	4757 (5.60)	**5.44 (5.07-5.84)**	**5.17 (4.82-5.55)**
**Acute respiratory complications**						
Comparators	45 (0.040)	1.0 (reference)	1.0 (reference)	122 (0.14)	1.0 (reference)	1.0 (reference)
Patients with COVID-19	115 (0.10)	**4.75 (3.08-7.33)**	**4.32 (2.73-6.83)**	924 (1.09)	**7.57 (6.27-9.14)**	**6.51 (5.38-7.87)**

CCI, Charlson comorbidity index; CI, confidence interval; HR, hazard ratio.
Bold indicates that hazard ratio is statistically significant (P<0.05; log-rank test).
***Model 1 and 3:** Adjusted for age (20–39, 40–59, and ≥60 years) and sex.
†**Model 2:** Adjusted for age (20–39, 40–59, and ≥60 years); sex, household income (low income, middle income, and high income); region of residence (urban and rural); CCI score (0, 1, and ≥2); obesity (underweight [<18.5 kg/m^2], normal [18.5–22.9 kg/m^2]; overweight [23.0–24.9 kg/m^2], obese [≥25.0 kg/m^2], and unknown); blood pressure (systolic blood pressure <140 mmHg and diastolic blood pressure <90 mmHg, systolic blood pressure ≥140 mmHg or diastolic blood pressure ≥90 mmHg, and unknown); fasting blood glucose (<100, ≥100 mg/dL, and unknown); serum total cholesterol (<200, 200–239, ≥240 mg/dL, and unknown); glomerular filtration rate (<60, 60–89, ≥90 mL/min/1.73 m^2, and unknown); smoking status (never, former, current smoker, and unknown); alcoholic drinks (<1, 1–2, 3–4, ≥5 days per week, and unknown); aerobic physical activity (sufficient, insufficient, and unknown); previous history of cardiovascular disease, and chronic kidney disease; history of medication use for diabetes mellitus, dyslipidemia, and hypertension; and strain of SARS-CoV-2 (original and delta).

∥ **Model 4:** Adjusted for age (20–39, 40–59, and ≥60 years); sex; insurance status (insured and dependent); CCI score (0, 1, and ≥ 2); body mass index (underweight [<18.5 kg/m^2], normal [18.5–22.9 kg/m^2], overweight [23.0–25.0 kg/m^2], obese [≥25.0 kg/m^2], and unknown); blood pressure (systolic blood pressure <140 mmHg and diastolic blood pressure <90 mmHg, systolic blood pressure ≥140 mmHg or diastolic blood pressure ≥90 mmHg, and unknown); fasting blood glucose (<100, ≥100 mg/dL, and unknown); serum total cholesterol (<200, 200–239, ≥240 mg/dL, and unknown); glomerular filtration rate (<60, 60–89, ≥90 mL/min/1.73 m^2, and unknown); smoking status (non- and current smoker, and unknown); alcoholic drinks (rarely, sometimes, everyday, and unknown); aerobic physical activity (sufficient, insufficient, and unknown); previous history of cardiovascular disease, and chronic kidney disease; history of medication use for diabetes mellitus, dyslipidemia, and hypertension; and strain of SARS-CoV-2 (original and delta).

Table S7. Baseline characteristics for the overlap-weighted cohort in South Korea (main)

Characteristic	Main cohort (n=754,742)		
	COVID-19 (n=377,371)	Non-COVID-19 (n=377,371)	SMD
**Mean age (SD), *y***	47.6 (16.5)	46.9 (2.9)	0.053
**Age, *n (%)***			<0.001
20–39 y	136,554 (36.2)	136,554 (36.2)	
40–59 y	139,057 (36.9)	139,056 (36.9)	
≥60 y	101,760 (27.0)	101,761 (27.0)	
**Sex, *n (%)***			<0.001
Male	196,487 (52.1)	196,487 (52.1)	
Female	180,884 (47.9)	180,884 (47.9)	
**Region of residence, *n (%)***			<0.001
Urban	203,682 (54.0)	203,682 (54.0)	
Rural	173,689 (46.0)	173,689 (46.0)	
**Medical history, *n (%)***			
Cardiovascular disease	17,644 (4.7)	17,644 (4.7)	<0.001
Chronic kidney disease	8406 (2.2)	8405 (2.2)	<0.001
Medication use for diabetes	68,892 (18.3)	68,892 (18.3)	<0.001
Medication use for hyperlipidemia	57,510 (15.2)	57,510 (15.2)	<0.001
Medication use for hypertension	31,163 (8.3)	31,163 (8.3)	<0.001
**Unmatching covariates, *n (%)***[†]			
Charlson Comorbidity Index score			0.230
0	331,387 (87.8)	355,625 (94.2)	

1	29,236 (7.8)	11,726 (3.1)	
≥2	16,748 (4.4)	10,020 (2.7)	
Household income			<0.001
Low (0th–39th percentile)	174,656 (46.3)	174,656 (46.3)	
Middle (40th –79th percentile)	134,003 (35.5)	134,003 (35.5)	
High (80th–100th percentile)	68,712 (18.2)	68,712 (18.2)	
Body mass index			1.212
Underweight (<18.5 kg/m^2)	6581 (1.7)	13,600 (3.6)	
Normal (18.5-22.9 kg/m^2)	74,312 (19.7)	135,565 (35.9)	
Overweight (23.0-24.9 kg/m^2)	52,016 (13.8)	85,947 (22.8)	
Obese (≥25.0 kg/m^2)	91,218 (24.2)	142,215 (37.7)	
Unknown	153,244 (40.6)	45 (0.01)	
Blood pressure			1.181
SBP <140 mmHg and DBP <90 mmHg	190,480 (50.5)	329,221 (87.2)	
SBP ≥140mmHg or DBP ≥90 mmHg	32,542 (8.6)	47,369 (12.6)	
Unknown	154,349 (40.9)	781 (0.2)	
Fasting blood glucose			1.181
<100 mg/dL	133,935 (35.5)	234,987 (62.3)	
≥100 mg/dL	89,068 (23.6)	141,591 (37.5)	
Unknown	154,368 (40.9)	793 (0.2)	
Serum total cholesterol			0.395
<200 mg/dL	64,465 (17.1)	103,189 (27.3)	
200 to 239 mg/dL	37,829 (10.0)	61,589 (16.3)	
≥240 mg/dL	16,051 (4.3)	25,767 (6.8)	
Unknown	259,026 (68.6)	186,826 (49.5)	
Glomerular filtration rate			1.180
<60 mL/min/1.73 m^2	8176 (2.2)	10,228 (2.7)	

60 to 89 mL/min/1.73 m^2	98,133 (26.0)	159,110 (42.2)	
≥90 mL/min/1.73 m^2	116,544 (30.9)	206,880 (54.8)	
Unknown	154,518 (41.0)	1153 (0.3)	
Smoking status			1.191
Never	147,844 (39.2)	239,173 (63.4)	
Former	40,867 (10.8)	59,401 (15.7)	
Current	35,423 (9.4)	78,677 (20.9)	
Unknown	153,237 (40.6)	120 (0.03)	
Alcohol consumption			1.156
<1 day/week	131,356 (34.8)	224,829 (59.6)	
1 to 2 days/week	62,664 (16.6)	107,501 (28.5)	
3 to 4 days/week	22,326 (5.9)	33,901 (9.0)	
≥5 days/week	7773 (2.1)	11,015 (2.9)	
Unknown	153,252 (40.6)	125 (0.03)	
Aerobic physical activity			1.179
Insufficient	114,084 (30.2)	187,416 (49.7)	
Sufficient	109,951 (29.1)	189,781 (50.3)	
Unknown	153,336 (40.6)	174 (0.1)	
Strain of SARS-CoV-2			0.003
Original	115,269 (30.6)	115,831 (30.7)	
Delta	262,102 (69.5)	261,540 (69.3)	

DBP, diastolic blood pressure; SARS-CoV-2, severe acute respiratory syndrome coronavirus 2; SBP, systolic blood pressure; SD, standard deviation; SMD, standardized mean difference.

* An SMD <0.1 indicates no significant imbalance. All SMDs were <0.1 in the propensity score–matched cohorts.

† Unmatched covariates were included as adjustment factors in statistical analyses.

Table S8. Baseline characteristics for the overlap-weighted cohort in Japan (replication)

	Replication cohort (n=1,276,694)		
**Characteristic**	COVID-19 (n=638,347)	General population (n=638,347)	SMD*
**Mean age (SD), *y***	44.4 (10.6)	44.8 (5.2)	0.050
**Age, *n (%)***			<0.001
20–39 y	227,172 (35.6)	227,172 (35.6)	
40–59 y	339,533 (53.2)	339,533 (53.2)	
≥60 y	71,642 (11.2)	71,642 (11.2)	
**Sex, *n (%)***			<0.001
Male	397,791 (62.3)	397,791 (62.3)	
Female	240,556 (37.7)	240,556 (37.7)	
**Insurance status, *n (%)***			<0.001
Insured	572,607 (89.7)	572,607 (89.7)	
Dependent	65,740 (10.3)	65,740 (10.3)	
**Medical history, *n (%)***			
Cardiovascular disease	47,750 (7.5)	47,750 (7.5)	<0.001
Chronic kidney disease	25,451 (4.0)	25,451 (4.0)	<0.001

Medication use for diabetes	16,160 (2.5)	16,160 (2.5)	<0.001
Medication use for hyperlipidemia	40,639 (6.4)	40,639 (6.4)	<0.001
Medication use for hypertension	54,908 (8.6)	54,908 (8.6)	<0.001
**Unmatching covariates, *n (%)*** [†]			
Charlson Comorbidity Index score			0.199
0	611,433 (95.8)	631,656 (99.0)	
1	8822 (1.4)	2336 (0.4)	
≥2	18,092 (2.8)	4355 (0.7)	
Body mass index			0.023
Underweight (<18.5 kg/m^2)	340,250 (53.3)	346,047 (54.2)	
Normal (18.5-22.9 kg/m^2)	122,190 (19.1)	122,020 (19.1)	
Overweight (23.0-24.9 kg/m^2)	137,212 (21.5)	134,235 (21.0)	
Obese (≥25.0 kg/m^2)	37,728 (5.9)	35,140 (5.5)	
Unknown	967 (0.2)	905 (0.1)	
Blood pressure			0.041
SBP <140 mmHg and DBP <90 mmHg	551,836 (86.5)	544,450 (85.3)	
SBP ≥140mmHg or DBP ≥90 mmHg	38,184 (6.0)	42,382 (6.6)	
Unknown	48,327 (7.6)	51,515 (8.1)	

Fasting blood glucose			0.028
<100 mg/dL	408,131 (63.9)	404,033 (63.3)	
≥100 mg/dL	131,693 (20.6)	133,988 (21.0)	
Unknown	98,523 (15.4)	100,326 (15.7)	
Serum total cholesterol			0.042
<200 mg/dL	295,814 (46.3)	282,972 (44.3)	
200 to 239 mg/dL	230,730 (36.1)	234,567 (36.8)	
≥240 mg/dL	100,841 (15.8)	106,454 (16.7)	
Unknown	10,962 (1.7)	14,354 (2.3)	
Glomerular filtration rate			0.038
<60 mL/min/1.73 m^2	3577 (0.6)	2301 (0.4)	
60 to 89 mL/min/1.73 m^2	60,619 (9.5)	61,394 (9.6)	
≥90 mL/min/1.73 m^2	347,601 (54.5)	340,887 (53.4)	
Unknown	226,550 (35.5)	233,765 (36.6)	
Smoking status			0.062
Non-smoker	145,103 (22.7)	152,628 (23.9)	
Smoker	470,406 (73.7)	462,568 (72.5)	
Unknown	22,838 (3.6)	23,151 (3.6)	

Alcohol consumption			0.027
Everyday	131,967 (20.7)	130,385 (20.4)	
Sometimes	210,682 (33.0)	208,601 (32.7)	
Rarely	249,294 (39.1)	253,317 (39.7)	
Unknown	46,404 (7.3)	46,044 (7.2)	
Aerobic physical activity			0.027
Insufficient	130,291 (20.4)	138,213 (21.7)	
Sufficient	450,219 (70.5)	441,711 (69.2)	
Unknown	57,837 (9.1)	58,423 (9.2)	
Strain of SARS-CoV-2			0.007
Original	268,315 (42.0)	270,612 (42.4)	
Delta	370,032 (58.0)	367,735 (57.6)	

DBP, diastolic blood pressure; SARS-CoV-2, severe acute respiratory syndrome coronavirus 2; SBP, systolic blood pressure; SD, standard deviation; SMD, standardized mean difference.

* An SMD <0.1 indicates no significant imbalance. All SMDs were <0.1 in the propensity score–matched cohorts.

† Unmatched covariates were included as adjustment factors in statistical analyses.

Table S9. Overlap-weighted HR (95% CI) for the **post-acute respiratory sequelae** or **acute respiratory complications** subtypes following COVID-19 diagnosis of patients in binational cohorts (COVID-19 vs. general population) in South Korea (main) and Japan (replication)

	Main cohort (n=754,742)			Replication cohort (n=1,276,694)		
		HR (95% CI)			HR (95% CI)	
	Events, *n (%)*	Model 1*	Model 2†	Events, *n (%)*	Model 3*	Model 4‖
**Post-acute respiratory sequelae**						
Comparators	3008 (0.80)	1.0 (reference)	1.0 (reference)	10,645 (1.67)	1.0 (reference)	1.0 (reference)
Patients with COVID-19	4735 (1.25)	**1.62 (1.55-1.70)**	**1.68 (1.60-1.77)**	35,451 (5.55)	**3.45 (3.38-3.53)**	**3.26 (3.19-3.33)**
**Chronic respiratory failure**						
Comparators	3 (0.00079)	1.0 (reference)	1.0 (reference)	40 (0.0063)	1.0 (reference)	1.0 (reference)
Patients with COVID-19	30 (0.0079)	**10.30 (3.18-33.35)**	**10.84 (3.21-36.64)**	565 (0.089)	**14.00 (10.17-19.27)**	**8.66 (6.27-11.97)**
**Pulmonary hypertension**						
Comparators	2 (0.00053)	1.0 (reference)	1.0 (reference)	43 (0.0067)	1.0 (reference)	1.0 (reference)
Patients with COVID-19	4 (0.0011)	2.43 (0.39-15.22)	0.47 (0.01-28.71)	175 (0.027)	**4.10 (2.94-5.72)**	**3.05 (2.17-4.28)**
**Sleep apnea**						
Comparators	209 (0.065)	1.0 (reference)	1.0 (reference)	1883 (0.30)	1.0 (reference)	1.0 (reference)
Patients with COVID-19	230 (0.061)	1.12 (0.93-1.35)	1.17 (0.95-1.45)	4210 (0.66)	**2.26 (2.14-2.39)**	**2.16 (2.05-2.29)**
**COPD**						
Comparators	2059 (0.55)	1.0 (reference)	1.0 (reference)	3046 (0.48)	1.0 (reference)	1.0 (reference)
Patients with COVID-19	3052 (0.81)	**1.52 (1.44-1.61)**	**1.54 (1.45-1.64)**	12,484 (1.96)	**4.17 (4.01-4.34)**	**3.91 (3.76-4.07)**
**Emphysema**						
Comparators	72 (0.019)	1.0 (reference)	1.0 (reference)	431 (0.068)	1.0 (reference)	1.0 (reference)
Patients with COVID-19	103 (0.027)	**1.50 (1.11-2.02)**	**1.52 (1.09-2.10)**	1719 (0.27)	**4.00 (3.60-4.44)**	**3.44 (3.10-3.83)**

**Asthma**						
Comparators	706 (0.19)	1.0 (reference)	1.0 (reference)	5877 (0.92)	1.0 (reference)	1.0 (reference)
Patients with COVID-19	1189 (0.32)	**1.72 (1.57-1.89)**	**1.88 (1.70-2.08)**	20,135 (3.15)	**3.50 (3.40-3.60)**	**3.41 (3.31-3.51)**
**Pulmonary sarcoidosis**						
Comparators	4 (0.0011)	1.0 (reference)	1.0 (reference)	267 (0.042)	1.0 (reference)	1.0 (reference)
Patients with COVID-19	4 (0.0011)	0.95 (0.24-3.85)	1.53 (0.37-6.30)	1021 (0.16)	**3.85 (3.36-4.40)**	**3.49 (3.04-3.99)**
**Interstitial lung disease**						
Comparators	35 (0.0093)	1.0 (reference)	1.0 (reference)	805 (0.13)	1.0 (reference)	1.0 (reference)
Patients with COVID-19	333 (0.088)	**9.85 (6.96-13.94)**	**10.57 (7.42-15.06)**	4913 (0.77)	**6.16 (5.72-6.64)**	**4.79 (4.44-5.17)**
**Acute respiratory complications**						
Comparators	88 (0.023)	1.0 (reference)	1.0 (reference)	685 (0.11)	1.0 (reference)	1.0 (reference)
Patients with COVID-19	812 (0.22)	**9.37 (7.52-11.68)**	**8.76 (6.98-10.98)**	7273 (1.14)	**10.64 (9.84-11.51)**	**9.09 (8.40-9.84)**
**Pneumocystis pneumonia**						
Comparators	1 (0.00026)	1.0 (reference)	1.0 (reference)	112 (0.018)	1.0 (reference)	1.0 (reference)
Patients with COVID-19	0 (0.00)	NA	NA	512 (0.080)	**4.55 (3.71-5.57)**	**3.34 (2.72-4.12)**
**Aspergillosis pneumonia**						
Comparators	2 (0.00053)	1.0 (reference)	1.0 (reference)	15 (0.0023)	1.0 (reference)	1.0 (reference)
Patients with COVID-19	31 (0.01)	**18.64 (3.91-88.82)**	**17.12 (3.50-83.88)**	171 (0.027)	**11.27 (6.68-19.02)**	**8.18 (4.82-13.89)**
**Pleural empyema**						
Comparators	3 (0.00079)	1.0 (reference)	1.0 (reference)	3 (0.00047)	1.0 (reference)	1.0 (reference)
Patients with COVID-19	8 (0.0021)	2.56 (0.68-9.58)	1.99 (0.44-8.96)	120 (0.018)	**35.48 (12.07-104.32)**	**29.66 (10.06-87.44)**
**Lung abscess**						
Comparators	5 (0.0013)	1.0 (reference)	1.0 (reference)	3 (0.00047)	1.0 (reference)	1.0 (reference)

Patients with COVID-19	8 (0.0021)	1.56 (0.51-4.82)	1.37 (0.36-5.26)	153 (0.024)	**48.16 (15.89-145.97)**	**44.01 (14.49-133.64)**
**Pneumothorax**						
Comparators	17 (0.0045)	1.0 (reference)	1.0 (reference)	121 (0.019)	1.0 (reference)	1.0 (reference)
Patients with COVID-19	88 (0.023)	**5.29 (3.15-8.90)**	**5.16 (2.99-8.89)**	751 (0.12)	**6.19 (5.11-7.50)**	**5.85 (4.82-7.09)**
**Acute respiratory failure**						
Comparators	4 (0.0011)	1.0 (reference)	1.0 (reference)	319 (0.050)	1.0 (reference)	1.0 (reference)
Patients with COVID-19	487 (0.13)	**120.86 (45.63-320.13)**	**106.37 (40.03-282.65)**	4741 (0.74)	**14.86 (13.27-16.64)**	**12.6 (11.25-14.12)**
**Pulmonary embolism**						
Comparators	56 (0.015)	1.0 (reference)	1.0 (reference)	130 (0.020)	1.0 (reference)	1.0 (reference)
Patients with COVID-19	195 (0.052)	**3.48 (2.59-4.68)**	**3.64 (2.66-4.98)**	1290 (0.20)	**9.88 (8.25-11.82)**	**8.21 (6.85-9.84)**

CCI, Charlson comorbidity index; CI, confidence interval; COPD, chronic obstructive pulmonary disease; NA, not available; OR, odds ratio; SARS-CoV-2, severe acute respiratory syndrome coronavirus 2.

Bold indicates that hazard ratio is statistically significant (P<0.05; log-rank test).

***Model 1 and 3:** Adjusted for age (20–39, 40–59, and ≥60 years) and sex.

†**Model 2:** Adjusted for age (20–39, 40–59, and ≥60 years); sex, household income (low income, middle income, and high income); region of residence (urban and rural); CCI score (0, 1, and ≥2); obesity (underweight [<18.5 kg/m^2], normal [18.5–22.9 kg/m^2]; overweight [23.0–24.9 kg/m^2], obese [≥25.0 kg/m^2], and unknown); blood pressure (systolic blood pressure <140 mmHg and diastolic blood pressure <90 mmHg, systolic blood pressure ≥140 mmHg or diastolic blood pressure ≥90 mmHg, and unknown); fasting blood glucose (<100, ≥100 mg/dL, and unknown); serum total cholesterol (<200, 200–239, ≥240 mg/dL, and unknown); glomerular filtration rate (<60, 60–89, ≥90 mL/min/1.73 m^2, and unknown); smoking status (never, former, current smoker, and unknown); alcoholic drinks (<1, 1–2, 3–4, ≥5 days per week, and unknown); aerobic physical activity (sufficient, insufficient, and unknown); previous history of cardiovascular disease, and chronic kidney disease; history of medication use for diabetes mellitus, dyslipidemia, and hypertension; and strain of SARS-CoV-2 (original and delta).

‖**Model 4 (replication):** Adjusted for age (20–39, 40–59, and ≥60 years); sex; insurance status (insured and dependent); CCI score (0, 1, and ≥ 2); body mass index (underweight [<18.5 kg/m^2], normal [18.5–22.9 kg/m^2], overweight [23.0–25.0 kg/m^2], obese [≥25.0 kg/m^2], and

unknown); blood pressure (systolic blood pressure <140 mmHg and diastolic blood pressure <90 mmHg, systolic blood pressure ≥140 mmHg or diastolic blood pressure ≥90 mmHg, and unknown); fasting blood glucose (<100, ≥100 mg/dL, and unknown); serum total cholesterol (<200, 200–239, ≥240 mg/dL, and unknown); glomerular filtration rate (<60, 60–89, ≥90 mL/min/1.73 m^2, and unknown); smoking status (non- and current smoker, and unknown); alcoholic drinks (rarely, sometimes, everyday, and unknown); aerobic physical activity (sufficient, insufficient, and unknown); previous history of cardiovascular disease, and chronic kidney disease; history of medication use for diabetes mellitus, dyslipidemia, and hypertension; and strain of SARS-CoV-2 (original and delta).

Table S10. HR (95% CI) for the **post-acute respiratory sequelae** or **acute respiratory complications** subtypes after SARS-CoV-2 infection in the propensity score-matched cohorts (COVID-19 vs. influenza) in South Korea (main) and Japan (replication)

**Cohort**	**South Korea**			**Japan**		
	COVID-19 *vs.* influenza (n=223,000)			COVID-19 *vs.* influenza (n=169,924)		
		HR (95% CI)			HR (95% CI)	
	Events, *n (%)*	Model 1*	Model 2†	Events, *n (%)*	Model 3*	Model 4‖
**Post-acute respiratory sequelae**						
**Chronic respiratory failure**						
Comparators (general population or patients with influenza)	3 (0.0027)	1.0 (reference)	1.0 (reference)	3 (0.0035)	1.0 (reference)	1.0 (reference)
Patients with COVID-19	12 (0.011)	**4.19 (1.18-14.86)**	**4.82 (1.28-18.12)**	77 (0.091)	**25.55 (8.06-80.95)**	**16.15 (5.04-51.80)**
**Pulmonary hypertension**						
Comparators	0 (0.00)	1.0 (reference)	1.0 (reference)	4 (0.0047)	1.0 (reference)	1.0 (reference)
Patients with COVID-19	0 (0.00)	NA	NA	28 (0.033)	**6.97 (2.44-19.87)**	**5.59 (1.93-16.14)**
**Sleep apnea**						
Comparators	23 (0.021)	1.0 (reference)	1.0 (reference)	88 (0.10)	1.0 (reference)	1.0 (reference)
Patients with COVID-19	49 (0.044)	**4.26 (2.26-8.01)**	**4.12 (2.09-8.12)**	549 (0.65)	**6.26 (5.00-7.84)**	**5.95 (4.74-7.46)**
**COPD**						
Comparators	734 (0.66)	1.0 (reference)	1.0 (reference)	271 (0.32)	1.0 (reference)	1.0 (reference)
Patients with COVID-19	994 (0.89)	**1.48 (1.34-1.63)**	**1.60 (1.44-1.79)**	1703 (2.00)	0.99 (0.91-1.09)	1.01 (0.91-1.12)
**Emphysema**						
Comparators	19 (0.017)	1.0 (reference)	1.0 (reference)	31 (0.036)	1.0 (reference)	1.0 (reference)
Patients with COVID-19	33 (0.030)	**1.96 (1.10-3.47)**	**1.99 (1.06-3.74)**	232 (0.27)	**7.47 (5.14-10.87)**	**6.46 (4.43-9.43)**
**Asthma**						

Comparators	337 (0.30)	1.0 (reference)	1.0 (reference)	477 (0.56)	1.0 (reference)	1.0 (reference)
Patients with COVID-19	421 (0.38)	**1.37 (1.18-1.58)**	**1.45 (1.23-1.71)**	2672 (3.14)	**5.72 (5.19-6.30)**	**5.59 (5.07-6.16)**
**Pulmonary sarcoidosis**						
Comparators	2 (0.0018)	1.0 (reference)	1.0 (reference)	25 (0.029)	1.0 (reference)	1.0 (reference)
Patients with COVID-19	2 (0.0018)	2.07 (0.19-22.84)	3.24 (0.29-36.47)	127 (0.15)	**5.08 (3.31-7.81)**	**4.52 (2.93-6.96)**
**Interstitial lung disease**						
Comparators	32 (0.029)	1.0 (reference)	1.0 (reference)	67 (0.079)	1.0 (reference)	1.0 (reference)
Patients with COVID-19	94 (0.084)	**3.25 (2.15-4.90)**	**3.37 (2.19-5.19)**	679 (0.80)	**10.19 (7.93-13.1)**	**8.26 (6.42-10.64)**
**Acute respiratory complications**						
**Pneumocystis pneumonia**						
Comparators	0 (0.00)	1.0 (reference)	1.0 (reference)	18 (0.021)	1.0 (reference)	1.0 (reference)
Patients with COVID-19	0 (0.00)	NA	NA	66 (0.078)	**3.65 (2.17-6.14)**	**2.85 (1.68-4.85)**
**Aspergillosis pneumonia**						
Comparators	0 (0.00)	1.0 (reference)	1.0 (reference)	4 (0.0047)	1.0 (reference)	1.0 (reference)
Patients with COVID-19	9 (0.0081)	NA	NA	17 (0.020)	**4.21 (1.42-12.52)**	**3.49 (1.16-10.54)**
**Pleural empyema**						
Comparators	2 (0.0018)	1.0 (reference)	1.0 (reference)	0 (0.00)	1.0 (reference)	1.0 (reference)
Patients with COVID-19	0 (0.00)	NA	NA	18 (0.021)	NA	NA
**Lung abscess**						
Comparators	6 (0.0054)	1.0 (reference)	1.0 (reference)	6 (0.0071)	1.0 (reference)	1.0 (reference)
Patients with COVID-19	0 (0.00)	NA	NA	18 (0.021)	**2.98 (1.18-7.50)**	2.25 (0.88-5.80)
**Pneumothorax**						
Comparators	18 (0.016)	1.0 (reference)	1.0 (reference)	22 (0.026)	1.0 (reference)	1.0 (reference)
Patients with COVID-19	10 (0.0090)	1.05 (0.44-2.53)	1.02 (0.37-2.81)	114 (0.13)	**5.17 (3.28-8.16)**	**4.92 (3.11-7.77)**
**Acute respiratory failure**						
Comparators	2 (0.0018)	1.0 (reference)	1.0 (reference)	58 (0.068)	1.0 (reference)	1.0 (reference)

Patients with COVID-19	67 (0.060)	NA	NA	583 (0.69)	**10.04 (7.67-13.15)**	**8.44 (6.43-11.07)**
**Pulmonary embolism**						
Comparators	18 (0.06)	1.0 (reference)	1.0 (reference)	18 (0.021)	1.0 (reference)	1.0 (reference)
Patients with COVID-19	29 (0.026)	**2.94 (1.43-6.03)**	**3.27 (1.53-7.02)**	175 (0.21)	**9.68 (5.96-15.73)**	**7.96 (4.88-12.98)**

CCI, Charlson comorbidity index; CI, confidence interval; COPD, chronic obstructive pulmonary disease; HR, hazard ratio; NA, not available. Bold indicates that hazard ratio is statistically significant (P<0.05; log-rank test).

***Model 1 and 3:** Adjusted for age (20–39, 40–59, and ≥60 years) and sex.

†**Model 2:** Adjusted for age (20–39, 40–59, and ≥60 years); sex, household income (low income, middle income, and high income); region of residence (urban and rural); CCI score (0, 1, and ≥2); obesity (underweight [<18.5 kg/m^2], normal [18.5–22.9 kg/m^2]; overweight [23.0–24.9 kg/m^2], obese [≥25.0 kg/m^2], and unknown); blood pressure (systolic blood pressure <140 mmHg and diastolic blood pressure <90 mmHg, systolic blood pressure ≥140 mmHg or diastolic blood pressure ≥90 mmHg, and unknown); fasting blood glucose (<100, ≥100 mg/dL, and unknown); serum total cholesterol (<200, 200–239, ≥240 mg/dL, and unknown); glomerular filtration rate (<60, 60–89, ≥90 mL/min/1.73 m^2, and unknown); smoking status (never, former, current smoker, and unknown); alcoholic drinks (<1, 1–2, 3–4, ≥5 days per week, and unknown); aerobic physical activity (sufficient, insufficient, and unknown); previous history of cardiovascular disease, and chronic kidney disease; history of medication use for diabetes mellitus, dyslipidemia, and hypertension; and strain of SARS-CoV-2 (original and delta).

‖**Model 4:** Adjusted for age (20–39, 40–59, and ≥60 years); sex; insurance status (insured and dependent); CCI score (0, 1, and ≥ 2); body mass index (underweight [<18.5 kg/m^2], normal [18.5–22.9 kg/m^2], overweight [23.0–25.0 kg/m^2], obese [≥25.0 kg/m^2], and unknown); blood pressure (systolic blood pressure <140 mmHg and diastolic blood pressure <90 mmHg, systolic blood pressure ≥140 mmHg or diastolic blood pressure ≥90 mmHg, and unknown); fasting blood glucose (<100, ≥100 mg/dL, and unknown); serum total cholesterol (<200, 200–239, ≥240 mg/dL, and unknown); glomerular filtration rate (<60, 60–89, ≥90 mL/min/1.73 m^2, and unknown); smoking status (non- and current smoker, and unknown); alcoholic drinks (rarely, sometimes, everyday, and unknown); aerobic physical activity (sufficient, insufficient, and unknown); previous history of cardiovascular disease, and chronic kidney disease; history of medication use for diabetes mellitus, dyslipidemia, and hypertension; and strain of SARS-CoV-2 (original and delta).

Table S11. Marginal predicted prevalence (percent; 95% CI) of general population *vs*. patients with COVID-19 in South Korea

	South Korea, percent (95% CI)			
	Post-acute respiratory sequelae		Acute respiratory complications	
	Model 1*	Model 2†	Model 1*	Model 2†
**Age group**				
**20-39 y**				
Comparators (general population)	0.83 (0.83-0.83)	0.83 (0.83-0.83)	0.01 (0.01-0.01)	0.01 (0.01-0.01)
Patients with COVID-19	1.05 (1.05-1.05)	1.04 (1.04-1.05)	0.05 (0.05-0.05)	0.05 (0.04-0.05)
**40-59 y**				
Comparators	0.74 (0.74-0.74)	0.74 (0.74-0.74)	0.01 (0.01-0.01)	0.01 (0.01-0.01)
Patients with COVID-19	1.28 (1.28-1.28)	1.28 (1.27-1.28)	0.11 (0.11-0.11)	0.11 (0.11-0.11)
**≥60 y**				
Comparators	1.00 (1.00-1.00)	0.97 (0.97-0.97)	0.03 (0.03-0.03)	0.03 (0.03-0.03)
Patients with COVID-19	1.82 (1.82-1.82)	1.75 (1.74-1.75)	0.37 (0.37-0.37)	0.37 (0.37-0.38)
**Sex**				
**Male**				
Comparators	0.79 (0.79-0.79)	0.77 (0.77-0.77)	0.02 (0.02-0.02)	0.02 (0.02-0.02)
Patients with COVID-19	1.28 (1.28-1.28)	1.25 (1.25-1.25)	0.17 (0.17-0.17)	0.17 (0.17-0.17)
**Female**				
Comparators	0.90 (0.90-0.90)	0.89 (0.89-0.89)	0.02 (0.02-0.02)	0.02 (0.02-0.02)
Patients with COVID-19	1.41 (1.41-1.41)	1.47 (1.46-1.48)	0.14 (0.14-0.14)	0.14 (0.14-0.15)
**Household income**				
**Low (0th–39th percentile)**				
Comparators	0.84 (0.84-0.84)	0.83 (0.83-0.83)	0.02 (0.02-0.02)	0.02 (0.02-0.02)
Patients with COVID-19	1.30 (1.30-1.30)	1.27 (1.27-1.28)	0.13 (0.13-0.13)	0.13 (0.13-0.13)

**Middle (40th –79th percentile)**				
Comparators	0.85 (0.85-0.85)	0.84 (0.84-0.84)	0.01 (0.01-0.01)	0.01 (0.01-0.01)
Patients with COVID-19	1.33 (1.33-1.33)	1.31 (1.31-1.31)	0.16 (0.16-0.16)	0.16 (0.16-0.17)
**High (80th–100th percentile)**				
Comparators	0.83 (0.83-0.83)	0.81 (0.81-0.81)	0.02 (0.02-0.02)	0.02 (0.02-0.02)
Patients with COVID-19	1.48 (1.47-1.48)	1.45 (1.44-1.45)	0.21 (0.20-0.21)	0.21 (0.20-0.21)
**Region of residence**				
**Urban**				
Comparators	0.81 (0.81-0.81)	0.80 (0.80-0.80)	0.02 (0.02-0.02)	0.02 (0.02-0.02)
Patients with COVID-19	1.32 (1.32-1.33)	1.30 (1.30-1.30)	0.13 (0.13-0.13)	0.13 (0.13-0.13)
**Rural**				
Comparators	0.88 (0.88-0.88)	0.87 (0.87-0.87)	0.01 (0.01-0.01)	0.01 (0.01-0.01)
Patients with COVID-19	1.36 (1.36-1.36)	1.34 (1.34-1.34)	0.19 (0.19-0.19)	0.19 (0.19-0.19)
**Charlson comorbidity index score**				
**0**				
Comparators	0.79 (0.79-0.79)	0.78 (0.78-0.78)	0.01 (0.01-0.01)	0.01 (0.01-0.01)
Patients with COVID-19	1.20 (1.20-1.20)	1.19 (1.19-1.19)	0.10 (0.10-0.10)	0.10 (0.10-0.10)
**≥1**				
Comparators	1.67 (1.67-1.67)	1.61 (1.61-1.61)	0.10 (0.10-0.10)	0.10 (0.10-0.10)
Patients with COVID-19	2.33 (2.33-2.34)	2.38 (2.35-2.41)	0.58 (0.58-0.59)	0.58 (0.58-0.59)

CI, confidence interval.

***Model 1:** Adjusted for age (20–39, 40–59, and ≥60 years) and sex.

†**Model 2:** Adjusted for age (20–39, 40–59, and ≥60 years); sex, household income (low income, middle income, and high income); region of residence (urban and rural); CCI score (0, 1, and ≥2); obesity (underweight [<18.5 kg/m^2], normal [18.5–22.9 kg/m^2]; overweight [23.0–24.9 kg/m^2], obese [≥25.0 kg/m^2], and unknown); blood pressure (systolic blood pressure <140 mmHg and diastolic blood pressure <90 mmHg, systolic blood pressure ≥140 mmHg or diastolic blood pressure ≥90 mmHg, and unknown); fasting blood glucose (<100, ≥100 mg/dL, and

unknown); serum total cholesterol (<200, 200–239, ≥240 mg/dL, and unknown); glomerular filtration rate (<60, 60–89, ≥90 mL/min/1.73 m^2, and unknown); smoking status (never, former, current smoker, and unknown); alcoholic drinks (<1, 1–2, 3–4, ≥5 days per week, and unknown); aerobic physical activity (sufficient, insufficient, and unknown); previous history of cardiovascular disease, and chronic kidney disease; history of medication use for diabetes mellitus, dyslipidemia, and hypertension; and strain of SARS-CoV-2 (original and delta).

Table S12. Marginal predicted prevalence (percent; 95% CI) of general population *vs*. patients with COVID-19 in Japan

	Japan, percent (95% CI)			
	Post-acute respiratory sequelae		Acute respiratory complications	
	Model 3*	Model 4‖	Model 3*	Model 4‖
**Age group**				
**20-39 y**				
Comparators (general population)	1.43 (1.43-1.44)	1.43 (1.43-1.43)	0.05 (0.04-0.05)	0.05 (0.05-0.05)
Patients with COVID-19	4.83 (4.82-4.83)	4.83 (4.82-4.83)	0.24 (0.24-0.24)	0.24 (0.24-0.24)
**40-59 y**				
Comparators	1.53 (1.53-1.54)	1.53 (1.52-1.53)	0.07 (0.07-0.08)	0.07 (0.07-0.07)
Patients with COVID-19	5.04 (5.04-5.04)	5.04 (5.04-5.05)	0.29 (0.29-0.29)	0.29 (0.29-0.29)
**≥60 y**				
Comparators	1.84 (1.83-1.84)	1.84 (1.84-1.84)	0.08 (0.08-0.08)	0.08 (0.08-0.08)
Patients with COVID-19	6.89 (6.88-6.89)	6.89 (6.87-6.91)	0.49 (0.49-0.49)	0.49 (0.49-0.49)
**Sex**				
**Male**				
Comparators	1.46 (1.46-1.47)	1.46 (1.46-1.47)	0.07 (0.06-0.07)	0.07 (0.07-0.08)
Patients with COVID-19	5.04 (5.03-5.04)	5.04 (5.03-5.04)	0.32 (0.32-0.32)	0.32 (0.32-0.32)
**Female**				
Comparators	1.62 (1.61-1.63)	1.62 (1.62-1.62)	0.06 (0.06-0.06)	0.06 (0.06-0.06)
Patients with COVID-19	5.35 (5.35-5.35)	5.35 (5.34-5.35)	0.24 (0.24-0.24)	0.24 (0.24-0.24)
**Charlson comorbidity index score**				
**0**				
Comparators	1.51 (1.50-1.52)	1.50 (1.49-1.51)	0.06 (0.06-0.07)	0.06 (0.06-0.06)

Patients with COVID-19	4.75 (4.74-4.75)	4.75 (4.74-4.75)	0.25 (0.24-0.25)	0.25 (0.25-0.25)
**≥1**				
Comparators	3.43 (3.43-3.43)	3.43 (3.42-3.43)	0.23 (0.22-0.24)	0.24 (0.24-0.24)
Patients with COVID-19	14.58 (14.56-14.59)	14.58 (14.56-14.60)	1.15 (1.15-1.16)	1.15 (1.15-1.16)

CI, confidence interval.

*__Model 3:__ Adjusted for age (20–39, 40–59, and ≥60 years) and sex.

‖ **Model 4:** Adjusted for age (20–39, 40–59, and ≥60 years); sex; insurance status (insured and dependent); CCI score (0, 1, and ≥ 2); body mass index (underweight [<18.5 kg/m^2], normal [18.5–22.9 kg/m^2], overweight [23.0–25.0 kg/m^2], obese [≥25.0 kg/m^2], and unknown); blood pressure (systolic blood pressure <140 mmHg and diastolic blood pressure <90 mmHg, systolic blood pressure ≥140 mmHg or diastolic blood pressure ≥90 mmHg, and unknown); fasting blood glucose (<100, ≥100 mg/dL, and unknown); serum total cholesterol (<200, 200–239, ≥240 mg/dL, and unknown); glomerular filtration rate (<60, 60–89, ≥90 mL/min/1.73 m^2, and unknown); smoking status (non- and current smoker, and unknown); alcoholic drinks (rarely, sometimes, everyday, and unknown); aerobic physical activity (sufficient, insufficient, and unknown); previous history of cardiovascular disease, and chronic kidney disease; history of medication use for diabetes mellitus, dyslipidemia, and hypertension; and strain of SARS-CoV-2 (original and delta).

Table S13. Propensity-score-matched subgroup analysis (COVID-19 vs. general population) of HR (95% CI) of **post-acute respiratory sequelae** or **acute respiratory complications** following COVID-19 diagnosis stratified by COVID-19 vaccination in South Korea

Variable	Events/total, *n/N (%)*	HR (95% CI) Model 1[*]	HR (95% CI) Model 2[†]
**Post-acute respiratory sequelae**			
**Number of SARS-CoV-2 vaccinations**			
Non-infected control	16,122/1,918,150 (0.84)	1.0 (reference)	1.0 (reference)
COVID-19 without SARS-CoV-2 vaccination	4331/200,539 (2.16)	**1.60 (1.55-1.66)**	**1.67 (1.61-1.74)**
COVID-19 after SARS-CoV-2 vaccination received once	493/38,852 (1.27)	**1.86 (1.70-2.04)**	**1.78 (1.62-1.96)**
COVID-19 after SARS-CoV-2 vaccination received twice or more	468/155,207 (0.30)	**1.81 (1.65-1.20)**	**1.68 (1.52-1.85)**
**Type of SARS-CoV-2 vaccinations**			
Non-infected control	16,122/1,918,150 (0.84)	1.0 (reference)	1.0 (reference)
COVID-19 without SARS-CoV-2 vaccination	4331/200,539 (2.16)	**1.60 (1.55-1.66)**	**1.67 (1.61-1.74)**
COVID-19 with viral vector SARS-CoV-2 vaccination	465/109,066 (0.43)	**1.79 (1.63-1.97)**	**1.66 (1.51-1.83)**
COVID-19 with mRNA SARS-CoV-2 vaccination	477/66,891 (0.71)	**1.93 (1.76-2.12)**	**1.85 (1.68-2.03)**
COVID-19 with both types of SARS-CoV-2 vaccination	19/18,102 (0.10)	1.19 (0.76-1.87)	1.10 (0.70-1.73)
**Acute respiratory complications**			
**Number of SARS-CoV-2 vaccinations**			
Non-infected control	311/1,918,150 (0.016)	1.0 (reference)	1.0 (reference)
COVID-19 without SARS-CoV-2 vaccination	415/200,539 (0.21)	**14.51 (12.52-16.83)**	**13.19 (11.07-15.71)**
COVID-19 after SARS-CoV-2 vaccination received once	56/38,852 (0.14)	**9.17 (6.90-12.19)**	**7.97 (5.96-10.67)**
COVID-19 after SARS-CoV-2 vaccination received twice or more	147/155,207 (0.09)	**5.06 (4.16-6.16)**	**4.23 (3.44-5.21)**

**Type of SARS-CoV-2 vaccinations**			
Non-infected control	311/1,918,150 (0.016)	1.0 (reference)	1.0 (reference)
COVID-19 without SARS-CoV-2 vaccination	415/200,539 (0.21)	**14.51 (12.52-16.83)**	**13.19 (11.07-15.71)**
COVID-19 with viral vector SARS-CoV-2 vaccination	79/109,066 (0.072)	**6.35 (4.95-8.14)**	**4.69 (3.63-6.07)**
COVID-19 with mRNA SARS-CoV-2 vaccination	117/66,891 (0.17)	**5.80 (4.67-7.20)**	**5.42 (4.33-6.78)**
COVID-19 with both types of SARS-CoV-2 vaccination	7/18,102 (0.039)	**2.81 (1.33-5.94)**	**2.33 (1.10-4.94)**

CCI, Charlson comorbidity index; CI, confidence interval; HR, hazard ratio; SARS-CoV-2, severe acute respiratory syndrome coronavirus 2. Bold indicates that hazard ratio is statistically significant (P<0.05; log-rank test).

§ Only 1:5–matched comparators in each patient group at the same index date were included to reduce immortal time bias.

***Model 1:** Adjusted for age (20–39, 40–59, and ≥60 years) and sex.

†**Model 2:** Adjusted for age (20–39, 40–59, and ≥60 years); sex, household income (low income, middle income, and high income); region of residence (urban and rural); CCI score (0, 1, and ≥2); obesity (underweight [<18.5 kg/m^2], normal [18.5–22.9 kg/m^2]; overweight [23.0–24.9 kg/m^2], obese [≥25.0 kg/m^2], and unknown); blood pressure (systolic blood pressure <140 mmHg and diastolic blood pressure <90 mmHg, systolic blood pressure ≥140 mmHg or diastolic blood pressure ≥90 mmHg, and unknown); fasting blood glucose (<100, ≥100 mg/dL, and unknown); serum total cholesterol (<200, 200–239, ≥240 mg/dL, and unknown); glomerular filtration rate (<60, 60–89, ≥90 mL/min/1.73 m^2, and unknown); smoking status (never, former, current smoker, and unknown); alcoholic drinks (<1, 1–2, 3–4, ≥5 days per week, and unknown); aerobic physical activity (sufficient, insufficient, and unknown); previous history of cardiovascular disease, and chronic kidney disease; history of medication use for diabetes mellitus, dyslipidemia, and hypertension; and strain of SARS-CoV-2 (original and delta).

Table S14. Time attenuation effect analysis (COVID-19 vs. influenza) of HR (95% CI) for the risk of the **post-acute respiratory sequelae** after SARS-CoV-2 infection in South Korea (main cohort) and Japan (replication cohort)

**Time**	COVID-19 *vs.* influenza	
	Main cohort†	Replication cohort‖
**Post-acute respiratory sequelae**		
<3 months	**2.28 (2.01-2.59)**	**8.88 (7.76-10.16)**
3–6 months	**1.24 (1.04-1.47)**	**3.81 (3.27-4.44)**
≥6 months	1.01 (0.83-1.23)	**3.65 (3.30-4.05)**

CCI, Charlson comorbidity index; CI, confidence interval; HR, hazard ratio; SARS-CoV-2, severe acute respiratory syndrome coronavirus 2. Bold indicates that hazard ratio is statistically significant (P<0.05; log-rank test).

† **Adjusted HR (main):** Adjusted for age (20–39, 40–59, and ≥60 years); sex, household income (low income, middle income, and high income); region of residence (urban and rural); CCI score (0, 1, and ≥2); obesity (underweight [<18.5 kg/m^2], normal [18.5–22.9 kg/m^2]; overweight [23.0–24.9 kg/m^2], obese [≥25.0 kg/m^2], and unknown); blood pressure (systolic blood pressure <140 mmHg and diastolic blood pressure <90 mmHg, systolic blood pressure ≥140 mmHg or diastolic blood pressure ≥90 mmHg, and unknown); fasting blood glucose (<100, ≥100 mg/dL, and unknown); serum total cholesterol (<200, 200–239, ≥240 mg/dL, and unknown); glomerular filtration rate (<60, 60–89, ≥90 mL/min/1.73 m^2, and unknown); smoking status (never, former, current smoker, and unknown); alcoholic drinks (<1, 1–2, 3–4, ≥5 days per week, and unknown); aerobic physical activity (sufficient, insufficient, and unknown); previous history of cardiovascular disease, and chronic kidney disease; history of medication use for diabetes mellitus, dyslipidemia, and hypertension; and strain of SARS-CoV-2 (original and delta).

‖ **Adjusted HR (replication):** Adjusted for age (20–39, 40–59, and ≥60 years); sex; insurance status (insured and dependent); CCI score (0, 1, and ≥ 2); body mass index (underweight [<18.5 kg/m^2], normal [18.5–22.9 kg/m^2], overweight [23.0–25.0 kg/m^2], obese [≥25.0 kg/m^2], and unknown); blood pressure (systolic blood pressure <140 mmHg and diastolic blood pressure <90 mmHg, systolic blood pressure ≥140 mmHg or diastolic blood pressure ≥90 mmHg, and unknown); fasting blood glucose (<100, ≥100 mg/dL, and unknown); serum total cholesterol (<200, 200–239, ≥240 mg/dL, and unknown); glomerular filtration rate (<60, 60–89, ≥90 mL/min/1.73 m^2, and unknown); smoking status (non- and current smoker, and unknown); alcoholic drinks (rarely, sometimes, everyday, and unknown); aerobic physical activity (sufficient, insufficient,

and unknown); previous history of cardiovascular disease, and chronic kidney disease; history of medication use for diabetes mellitus, dyslipidemia, and hypertension; and strain of SARS-CoV-2 (original and delta).

Table S15. Baseline characteristics for 1:5 propensity score–matched cohort (positive for COVID-19 vs. negative for COVID-19 after the PCR test) in South Korea

**Characteristic**	COVID-19 *vs.* general population (n=492,774)		
	COVID-19 (n=82,938)	General population (n=409,836)	SMD*
**Mean age (SD),** ***y***	50.3 (18.0)	48.8 (15.1)	0.094
**Age,** ***n (%)***			<0.001
20–39 y	27150 (32.7)	135507 (33.1)	
40–59 y	28084 (33.9)	139600 (34.1)	
≥60 y	27704 (33.4)	134729 (32.9)	
**Sex,** ***n (%)***			0.007
Male	41715 (50.3)	205410 (50.1)	
Female	41223 (49.7)	204426 (49.9)	
**Region of residence,** ***n (%)***			<0.001
Urban	42950 (51.8)	212152 (51.8)	
Rural	39988 (48.2)	197684 (48.2)	
**Medical history,** ***n (%)***			
Cardiovascular disease	6367 (7.7)	30945 (7.6)	0.005
Chronic kidney disease	3127 (3.8)	14807 (3.6)	0.008
Medication use for diabetes	19346 (23.3)	94165 (23.0)	0.008
Medication use for hyperlipidemia	15948 (19.2)	78045 (19.0)	0.005
Medication use for hypertension	9556 (11.5)	45993 (11.2)	0.009
**Unmatching covariates,** ***n (%)*** †			

Charlson Comorbidity Index score			0.172
0	65267 (78.7)	337680 (82.4)	
1	10720 (12.9)	33672 (8.2)	
≥2	6951 (8.4)	38484 (9.4)	
Household income			<0.001
Low (0th–39th percentile)	36117 (43.6)	178323 (43.5)	
Middle (40th –79th percentile)	30486 (36.8)	150810 (36.8)	
High (80th–100th percentile)	16335 (19.7)	80703 (19.7)	
Body mass index			0.965
Underweight (<18.5 kg/m^2)	1478 (1.8)	13305 (3.3)	
Normal (18.5-22.9 kg/m^2)	16334 (19.7)	137555 (33.6)	
Overweight (23.0-24.9 kg/m^2)	11688 (14.1)	90952 (22.2)	
Obese (≥25.0 kg/m^2)	21462 (25.9)	157427 (38.4)	
Unknown	31976 (38.6)	10597 (2.6)	
Blood pressure			0.986
SBP <140 mmHg and DBP <90 mmHg	42980 (51.8)	345942 (84.4)	
SBP ≥140mmHg or DBP ≥90 mmHg	7576 (9.1)	51749 (12.6)	
Unknown	32382 (39.0)	12145 (3.0)	
Fasting blood glucose			0.986
<100 mg/dL	29647 (35.8)	240935 (58.8)	
≥100 mg/dL	20904 (25.2)	156723 (38.2)	
Unknown	32387 (39.1)	12178 (3.0)	
Serum total cholesterol			0.371
<200 mg/dL	15373 (18.5)	117059 (28.6)	
200 to 239 mg/dL	8155 (9.8)	64787 (15.8)	
≥240 mg/dL	3458 (4.2)	26592 (6.5)	

Unknown	55952 (67.5)	201398 (49.1)	
Glomerular filtration rate			0.989
<60 mL/min/1.73 m^2	2538 (3.1)	15911 (3.9)	
60 to 89 mL/min/1.73 m^2	22089 (26.6)	165622 (40.4)	
≥90 mL/min/1.73 m^2	25887 (31.2)	215705 (52.6)	
Unknown	32424 (39.1)	12598 (3.1)	
Smoking status			1.040
Never	34178 (41.2)	256807 (62.7)	
Former	9441 (11.4)	64616 (15.8)	
Current	7351 (8.9)	77779 (19.0)	
Unknown	31968 (38.5)	10634 (2.6)	
Alcohol consumption			0.964
<1 day/week	31148 (37.6)	247938 (60.5)	
1 to 2 days/week	13581 (16.4)	106578 (26)	
3 to 4 days/week	4614 (5.6)	33186 (8.1)	
≥5 days/week	1624 (2.0)	11508 (2.8)	
Unknown	31971 (38.6)	10626 (2.6)	
Aerobic physical activity			0.962
Insufficient	26430 (31.9)	202726 (49.5)	
Sufficient	24521 (29.6)	196414 (47.9)	
Unknown	31987 (38.6)	10696 (2.6)	
Strain of SARS-CoV-2			0.004
Original	32636 (39.4)	162008 (39.5)	
Delta	50302 (60.7)	247828 (60.5)	

DBP, diastolic blood pressure; PCR, polymerase chain reaction; SARS-CoV-2, severe acute respiratory syndrome coronavirus 2. SBP, systolic

blood pressure; SD, standard deviation; SMD, standardized mean difference.

* An SMD <0.1 indicates no significant imbalance. All SMDs were <0.1 in the propensity score–matched cohorts.

† Unmatched covariates were included as adjustment factors in statistical analyses.

Table S16. Stratification analysis for the risk of **post-acute respiratory sequelae** following COVID-19 in the propensity score matching cohorts (COVID-19 vs. general population) of South Korea (main)

	COVID-19 *vs.* general population (n=2,312,748)		
		HR (95% CI)	
	Events, *n (%)*	Model 1*	Model 2†
**Sex**			
**Male**			
Comparators (general population or patients with influenza)	7849 (0.79)	1.0 (reference)	1.0 (reference)
Patients with COVID-19	75 (1.28)	**1.67 (1.60-1.75)**	**1.64 (1.55-1.73)**
**Female**			
Comparators	8273 (0.90)	1.0 (reference)	1.0 (reference)
Patients with COVID-19	2666 (1.41)	**1.61 (1.54-1.68)**	**1.67 (1.59-1.76)**
**Age**			
**20–39 y**			
Comparators	5809 (0.83)	1.0 (reference)	1.0 (reference)
Patients with COVID-19	1501 (1.05)	**1.27 (1.20-1.35)**	**1.33 (1.23-1.44)**
**40–59 years**			
Comparators	5243 (0.74)	1.0 (reference)	1.0 (reference)
Patients with COVID-19	1861 (1.28)	**1.76 (1.66-1.85)**	**1.79 (1.68-1.89)**
**≥60 y**			
Comparators	5070 (1.0010)	1.0 (reference)	1.0 (reference)
Patients with COVID-19	1930 (1.82)	**1.96 (1.86-2.07)**	**1.85 (1.74-1.96)**
**Region of residence**			
**Rural**			
Comparators	8382 (0.81)	1.0 (reference)	1.0 (reference)
Patients with COVID-19	2821 (1.32)	**1.68 (1.61-1.75)**	**1.71 (1.63-1.80)**

**Urban**			
Comparators	7740 (0.88)	1.0 (reference)	1.0 (reference)
Patients with COVID-19	2471 (1.36)	**1.60 (1.53-1.67)**	**1.60 (1.51-1.68)**
**Income level**			
**Low (0th–39th percentile)**			
Comparators	7444 (0.84)	1.0 (reference)	1.0 (reference)
Patients with COVID-19	2370 (1.30)	**1.59 (1.52-1.67)**	**1.60 (1.51-1.69)**
**Middle (40th –79th percentile)**			
Comparators	5786 (0.85)	1.0 (reference)	1.0 (reference)
Patients with COVID-19	1861 (1.33)	**1.60 (1.52-1.69)**	**1.61 (1.51-1.71)**
**High (80th–100th percentile)**			
Comparators	2892 (0.83)	1.0 (reference)	1.0 (reference)
Patients with COVID-19	1061 (1.48)	**1.86 (1.73-1.99)**	**1.86 (1.72-2.02)**
**CCI score**			
**0 score**			
Comparators	14,263 (0.79)	1.0 (reference)	1.0 (reference)
Patients with COVID-19	4171 (1.20)	**1.59 (1.54-1.65)**	**1.72 (1.66-1.80)**
**≥1 score**			
Comparators	1859 (1.67)	1.0 (reference)	1.0 (reference)
Patients with COVID-19	1121 (2.33)	**1.31 (1.22-1.42)**	**1.39 (1.27-1.51)**
**BMI**			
**<18.5 kg/m^2**			
Comparators	549 (0.81)	1.0 (reference)	1.0 (reference)
Patients with COVID-19	89 (1.29)	**1.66 (1.32-2.07)**	**1.59 (1.27-2.00)**
**18.5-23.0 kg/m^2**			
Comparators	5575 (0.81)	1.0 (reference)	1.0 (reference)
Patients with COVID-19	989 (1.27)	**1.58 (1.48-1.69)**	**1.49 (1.39-1.60)**
**23.0-25.0 kg/m^2**			
Comparators	6362 (0.88)	1.0 (reference)	1.0 (reference)

Patients with COVID-19	1548 (1.63)	**1.83 (1.74-1.94)**	**1.72 (1.63-1.82)**
**≥25.0 kg/m²**			
Comparators	2 (0.90)	1.0 (reference)	1.0 (reference)
Patients with COVID-19	1822 (1.14)	1.10 (0.27-4.40)	5.28 (0.41-67.90)
**Unknown**			
Comparators	3634 (0.83)	1.0 (reference)	1.0 (reference)
Patients with COVID-19	844 (1.55)	**1.87 (1.73-2.01)**	**1.75 (1.62-1.89)**
**Smoking status**			
**Non-smoker**			
Comparators	10,215 (0.84)	1.0 (reference)	1.0 (reference)
Patients with COVID-19	2333 (1.51)	**1.79 (1.71-1.88)**	**1.70 (1.62-1.78)**
**Smoker**			
Comparators	5905 (0.84)	1.0 (reference)	1.0 (reference)
Patients with COVID-19	1138 (1.42)	**1.71 (1.60-1.82)**	**1.58 (1.48-1.69)**
**Unknown**			
Comparators	2 (0.35)	1.0 (reference)	1.0 (reference)
Patients with COVID-19	1821 (1.14)	3.88 (0.97-15.49)	0.23 (0.00-60.08)
**Alcohol consumption**			
**Non-drinker**			
Comparators	10,042 (0.88)	1.0 (reference)	1.0 (reference)
Patients with COVID-19	2140 (1.56)	**1.79 (1.70-1.87)**	**1.67 (1.60-1.75)**
**Drinker**			
Comparators	6077 (0.79)	1.0 (reference)	1.0 (reference)
Patients with COVID-19	1331 (1.37)	**1.73 (1.63-1.83)**	**1.63 (1.53-1.73)**
**Unknown**			
Comparators	3 (0.47)	1.0 (reference)	1.0 (reference)
Patients with COVID-19	1821 (1.14)	2.62 (0.84-8.12)	0.18 (0.00-24.24)
**Physical activity**			
**Insufficient physical activity**			

Comparators	8479 (0.88)	1.0 (reference)	1.0 (reference)
Patients with COVID-19	1773 (1.49)	**1.69 (1.60-1.77)**	**1.57 (1.49-1.65)**
**Sufficient physical activity**			
Comparators	7635 (0.80)	1.0 (reference)	1.0 (reference)
Patients with COVID-19	1698 (1.47)	**1.85 (1.75-1.95)**	**1.76 (1.67-1.85)**
**Unknown**			
Comparators	8 (0.92)	1.0 (reference)	1.0 (reference)
Patients with COVID-19	1821 (1.13)	1.48 (0.74-2.96)	0.09 (0.00-3.16)

BMI, body mass index; CCI, Charlson comorbidity index; CI, confidence interval; HR, hazard ratio; NA, not available; SARS-CoV-2, severe acute respiratory syndrome coronavirus 2.

Bold indicates that hazard ratio is statistically significant (P<0.05; log-rank test).

***Model 1:** Adjusted for age (20–39, 40–59, and ≥60 years) and sex.

†**Model 2 (main):** Adjusted for age (20–39, 40–59, and ≥60 years); sex, household income (low income, middle income, and high income); region of residence (urban and rural); CCI score (0, 1, and ≥2); obesity (underweight [<18.5 kg/m^2], normal [18.5–22.9 kg/m^2]; overweight [23.0–24.9 kg/m^2], obese [≥25.0 kg/m^2], and unknown); blood pressure (systolic blood pressure <140 mmHg and diastolic blood pressure <90 mmHg, systolic blood pressure ≥140 mmHg or diastolic blood pressure ≥90 mmHg, and unknown); fasting blood glucose (<100, ≥100 mg/dL, and unknown); serum total cholesterol (<200, 200–239, ≥240 mg/dL, and unknown); glomerular filtration rate (<60, 60–89, ≥90 mL/min/1.73 m^2, and unknown); smoking status (never, former, current smoker, and unknown); alcoholic drinks (<1, 1–2, 3–4, ≥5 days per week, and unknown); aerobic physical activity (sufficient, insufficient, and unknown); previous history of cardiovascular disease, and chronic kidney disease; history of medication use for diabetes mellitus, dyslipidemia, and hypertension; and strain of SARS-CoV-2 (original and delta).

Table S17. Stratification analysis for the risk of **post-acute respiratory sequelae** following COVID-19 in the propensity score matching cohorts of (COVID-19 vs. influenza) South Korea (main)

	COVID-19 *vs.* influenza (n=223,000)		
		HR (95% CI)	
	Events, *n (%)*	Model 1*	Model 2†
**Sex**			
**Male**			
Comparators (general population or patients with influenza)	515 (1.04)	1.0 (reference)	1.0 (reference)
Patients with COVID-19	581 (1.25)	**1.34 (1.19-1.52)**	**1.40 (1.21-1.61)**
**Female**			
Comparators	566 (0.91)	1.0 (reference)	1.0 (reference)
Patients with COVID-19	919 (1.41)	**1.69 (1.52-1.88)**	**1.82 (1.62-2.05)**
**Age**			
**20–39 y**			
Comparators	481 (1.20)	1.0 (reference)	1.0 (reference)
Patients with COVID-19	465 (1.09)	0.97 (0.85-1.11)	1.05 (0.89-1.23)
**40–59 years**			
Comparators	436 (0.82)	1.0 (reference)	1.0 (reference)
Patients with COVID-19	651 (1.28)	**1.68 (1.49-1.90)**	**1.75 (1.53-2.00)**
**≥60 y**			
Comparators	164 (0.91)	1.0 (reference)	1.0 (reference)
Patients with COVID-19	384 (2.11)	**2.91 (2.40-3.53)**	**3.15 (2.56-3.88)**
**Region of residence**			
**Rural**			
Comparators	448 (0.91)	1.0 (reference)	1.0 (reference)
Patients with COVID-19	624 (1.26)	**1.56 (1.38-1.77)**	**1.73 (1.51-1.99)**
**Urban**			

Comparators	633 (1.02)	1.0 (reference)	1.0 (reference)
Patients with COVID-19	876 (1.41)	**1.51 (1.36-1.68)**	**1.57 (1.39-1.76)**
**Income level**			
**Low (0th–39th percentile)**			
Comparators	454 (0.99)	1.0 (reference)	1.0 (reference)
Patients with COVID-19	671 (1.37)	**1.54 (1.36-1.74)**	**1.69 (1.47-1.94)**
**Middle (40th –79th percentile)**			
Comparators	432 (0.96)	1.0 (reference)	1.0 (reference)
Patients with COVID-19	596 (1.31)	**1.50 (1.32-1.70)**	**1.59 (1.38-1.83)**
**High (80th–100th percentile)**			
Comparators	195 (0.96)	1.0 (reference)	1.0 (reference)
Patients with COVID-19	233 (1.35)	**1.59 (1.31-1.94)**	**1.62 (1.30-2.02)**
**CCI score**			
**0 score**			
Comparators	988 (0.96)	1.0 (reference)	1.0 (reference)
Patients with COVID-19	1195 (1.20)	**1.39 (1.27-1.51)**	**1.52 (1.38-1.67)**
**≥1 score**			
Comparators	93 (1.11)	1.0 (reference)	1.0 (reference)
Patients with COVID-19	305 (2.58)	**2.34 (1.84-2.98)**	**2.75 (2.12-3.56)**
**BMI**			
**<18.5 kg/m^2**			
Comparators	35 (0.88)	1.0 (reference)	1.0 (reference)
Patients with COVID-19	26 (1.23)	1.62 (0.96-2.72)	**1.72 (1.01-2.92)**
**18.5-23.0 kg/m^2**			
Comparators	357 (0.90)	1.0 (reference)	1.0 (reference)
Patients with COVID-19	311 (1.36)	**1.67 (1.43-1.96)**	**1.68 (1.44-1.96)**
**23.0-25.0 kg/m^2**			
Comparators	235 (0.96)	1.0 (reference)	1.0 (reference)
Patients with COVID-19	233 (1.63)	**1.78 (1.48-2.13)**	**1.80 (1.50-2.17)**

**≥25.0 kg/m^2**			
Comparators	454 (1.04)	1.0 (reference)	1.0 (reference)
Patients with COVID-19	384 (1.53)	**1.56 (1.35-1.79)**	**1.52 (1.32-1.75)**
**Unknown**			
Comparators	0 (0.00)	1.0 (reference)	1.0 (reference)
Patients with COVID-19	546 (1.16)	N/A	N/A
**Smoking status**			
**Non-smoker**			
Comparators	717 (0.96)	1.0 (reference)	1.0 (reference)
Patients with COVID-19	681 (1.50)	**1.67 (1.50-1.86)**	**1.66 (1.49-1.85)**
**Smoker**			
Comparators	364 (1.00)	1.0 (reference)	1.0 (reference)
Patients with COVID-19	274 (1.45)	**1.59 (1.35-1.86)**	**1.59 (1.35-1.87)**
**Unknown**			
Comparators	0 (0.00)	1.0 (reference)	1.0 (reference)
Patients with COVID-19	545 (1.16)	N/A	N/A
**Alcohol consumption**			
**Non-drinker**			
Comparators	631 (0.93)	1.0 (reference)	1.0 (reference)
Patients with COVID-19	609 (1.58)	**1.87 (1.66-2.09)**	**1.84 (1.64-2.07)**
**Drinker**			
Comparators	450 (1.04)	1.0 (reference)	1.0 (reference)
Patients with COVID-19	346 (1.33)	**1.36 (1.18-1.57)**	**1.37 (1.19-1.58)**
**Unknown**			
Comparators	0 (0.00)	1.0 (reference)	1.0 (reference)
Patients with COVID-19	545 (1.16)	N/A	N/A
**Physical activity**			
**Insufficient physical activity**			
Comparators	554 (0.95)	1.0 (reference)	1.0 (reference)

Patients with COVID-19	507 (1.50)	**1.69 (1.49-1.91)**	**1.67 (1.48-1.89)**
**Sufficient physical activity**			
Comparators	526 (0.99)	1.0 (reference)	1.0 (reference)
Patients with COVID-19	448 (1.47)	**1.61 (1.41-1.83)**	**1.60 (1.41-1.82)**
**Unknown**			
Comparators	1 (2.44)	1.0 (reference)	1.0 (reference)
Patients with COVID-19	545 (1.16)	0.47 (0.07-3.37)	N/A

BMI, body mass index; CCI, Charlson comorbidity index; CI, confidence interval; HR, hazard ratio; NA, not available; SARS-CoV-2, severe acute respiratory syndrome coronavirus 2.

Bold indicates that hazard ratio is statistically significant (P<0.05; log-rank test).

***Model 1:** Adjusted for age (20–39, 40–59, and ≥60 years) and sex.

†**Model 2 (main):** Adjusted for age (20–39, 40–59, and ≥60 years); sex, household income (low income, middle income, and high income); region of residence (urban and rural); CCI score (0, 1, and ≥2); obesity (underweight [<18.5 kg/m^2], normal [18.5–22.9 kg/m^2]; overweight [23.0–24.9 kg/m^2], obese [≥25.0 kg/m^2], and unknown); blood pressure (systolic blood pressure <140 mmHg and diastolic blood pressure <90 mmHg, systolic blood pressure ≥140 mmHg or diastolic blood pressure ≥90 mmHg, and unknown); fasting blood glucose (<100, ≥100 mg/dL, and unknown); serum total cholesterol (<200, 200–239, ≥240 mg/dL, and unknown); glomerular filtration rate (<60, 60–89, ≥90 mL/min/1.73 m^2, and unknown); smoking status (never, former, current smoker, and unknown); alcoholic drinks (<1, 1–2, 3–4, ≥5 days per week, and unknown); aerobic physical activity (sufficient, insufficient, and unknown); previous history of cardiovascular disease, and chronic kidney disease; history of medication use for diabetes mellitus, dyslipidemia, and hypertension; and strain of SARS-CoV-2 (original and delta).

Table S18. Stratification analysis for the risk of **acute respiratory complications** following COVID-19 in the propensity score matching cohorts (COVID-19 vs. general population) of South Korea (main)

	COVID-19 *vs.* general population (n=2,312,748)		
		HR (95% CI)	
	Events, *n (%)*	Model 1*	Model 2†
**Sex**			
**Male**			
Comparators (general population or patients with influenza)	167 (0.017)	1.0 (reference)	1.0 (reference)
Patients with COVID-19	345 (0.17)	**10.08 (8.38-12.13)**	**8.72 (7.11-10.68)**
**Female**			
Comparators	144 (0.016)	1.0 (reference)	1.0 (reference)
Patients with COVID-19	273 (0.14)	**9.26 (7.56-11.33)**	**6.67 (5.28-8.43)**
**Age**			
**20–39 y**			
Comparators	61 (0.0087)	1.0 (reference)	1.0 (reference)
Patients with COVID-19	65 (0.045)	**5.23 (3.69-7.41)**	**5.85 (3.85-8.91)**
**40–59 years**			
Comparators	83 (0.012)	1.0 (reference)	1.0 (reference)
Patients with COVID-19	157 (0.11)	**9.26 (7.10-12.09)**	**7.14 (5.31-9.60)**
**≥60 y**			
Comparators	167 (0.033)	1.0 (reference)	1.0 (reference)
Patients with COVID-19	396 (0.37)	**11.55 (9.64-13.84)**	**8.69 (7.10-10.62)**
**Region of residence**			
**Rural**			
Comparators	185 (0.018)	1.0 (reference)	1.0 (reference)
Patients with COVID-19	271 (0.13)	**7.15 (5.93-8.62)**	**5.61 (4.52-6.96)**
**Urban**			

Comparators	126 (0.014)	1.0 (reference)	1.0 (reference)
Patients with COVID-19	347 (0.19)	**13.46 (10.97-16.50)**	**10.81 (8.64-13.51)**
**Income level**			
**Low (0th–39th percentile)**			
Comparators	142 (0.016)	1.0 (reference)	1.0 (reference)
Patients with COVID-19	241 (0.13)	**8.28 (6.73-10.19)**	**7.06 (5.58-8.94)**
**Middle (40th –79th percentile)**			
Comparators	99 (0.015)	1.0 (reference)	1.0 (reference)
Patients with COVID-19	229 (0.16)	**11.26 (8.90-14.26)**	**8.64 (6.65-11.22)**
**High (80th–100th percentile)**			
Comparators	70 (0.020)	1.0 (reference)	1.0 (reference)
Patients with COVID-19	148 (0.21)	**10.36 (7.80-13.77)**	**7.72 (5.61-10.61)**
**CCI score**			
**0 score**			
Comparators	197 (0.011)	1.0 (reference)	1.0 (reference)
Patients with COVID-19	338 (0.10)	**9.36 (7.85-11.16)**	**8.83 (7.25-10.76)**
**≥1 scores**			
Comparators	114 (0.10)	1.0 (reference)	1.0 (reference)
Patients with COVID-19	280 (0.58)	**5.60 (4.50-6.96)**	**5.85 (4.60-7.44)**
**BMI**			
**<18.5 kg/m^2**			
Comparators	13 (0.019)	1.0 (reference)	1.0 (reference)
Patients with COVID-19	11 (0.16)	**8.37 (3.73-18.78)**	**8.08 (3.57-18.32)**
**18.5-23.0 kg/m^2**			
Comparators	81 (0.012)	1.0 (reference)	1.0 (reference)
Patients with COVID-19	70 (0.090)	**7.22 (5.24-9.95)**	**6.15 (4.43-8.53)**
**23.0-25.0 kg/m^2**			
Comparators	71 (0.016)	1.0 (reference)	1.0 (reference)
Patients with COVID-19	85 (0.16)	**8.94 (6.52-12.25)**	**7.92 (5.75-10.91)**

**≥25.0 kg/m^2**			
Comparators	146 (0.020)	1.0 (reference)	1.0 (reference)
Patients with COVID-19	201 (0.21)	**9.51 (7.68-11.78)**	**8.25 (6.64-10.26)**
**Unknown**			
Comparators	0 (0.00)	1.0 (reference)	1.0 (reference)
Patients with COVID-19	251 (0.16)	N/A	N/A
**Smoking status**			
**Non-smoker**			
Comparators	189 (0.016)	1.0 (reference)	1.0 (reference)
Patients with COVID-19	239 (0.16)	**8.95 (7.40-10.84)**	**7.72 (6.35-9.38)**
**Smoker**			
Comparators	122 (0.017)	1.0 (reference)	1.0 (reference)
Patients with COVID-19	128 (0.16)	**8.50 (6.63-10.90)**	**7.56 (5.88-9.73)**
**Unknown**			
Comparators	0 (0.00)	1.0 (reference)	1.0 (reference)
Patients with COVID-19	251 (0.16)	N/A	N/A
**Alcohol consumption**			
**Non-drinker**			
Comparators	208 (0.018)	1.0 (reference)	1.0 (reference)
Patients with COVID-19	243 (0.18)	**8.88 (7.38-10.69)**	**7.75 (6.41-9.36)**
**Drinker**			
Comparators	103 (0.013)	1.0 (reference)	1.0 (reference)
Patients with COVID-19	124 (0.13)	**8.90 (6.85-11.56)**	**7.53 (5.77-9.83)**
**Unknown**			
Comparators	0 (0.00)	1.0 (reference)	1.0 (reference)
Patients with COVID-19	251 (0.16)	N/A	N/A
**Physical activity**			
**Insufficient physical activity**			
Comparators	154 (0.016)	1.0 (reference)	1.0 (reference)

Patients with COVID-19	212 (0.18)	**9.84 (7.99-12.12)**	**8.46 (6.84-10.46)**
**Sufficient physical activity**			
Comparators	157 (0.016)	1.0 (reference)	1.0 (reference)
Patients with COVID-19	155 (0.13)	**7.68 (6.15-9.59)**	**6.82 (5.45-8.55)**
**Unknown**			
Comparators	0 (0.00)	1.0 (reference)	1.0 (reference)
Patients with COVID-19	251 (0.16)	N/A	N/A

BMI, body mass index; CCI, Charlson comorbidity index; CI, confidence interval; HR, hazard ratio; NA, not available; SARS-CoV-2, severe acute respiratory syndrome coronavirus 2.

Bold indicates that hazard ratio is statistically significant (P<0.05; log-rank test).

***Model 1:** Adjusted for age (20–39, 40–59, and ≥60 years) and sex.

†**Model 2 (main):** Adjusted for age (20–39, 40–59, and ≥60 years); sex, household income (low income, middle income, and high income); region of residence (urban and rural); CCI score (0, 1, and ≥2); obesity (underweight [<18.5 kg/m^2], normal [18.5–22.9 kg/m^2]; overweight [23.0–24.9 kg/m^2], obese [≥25.0 kg/m^2], and unknown); blood pressure (systolic blood pressure <140 mmHg and diastolic blood pressure <90 mmHg, systolic blood pressure ≥140 mmHg or diastolic blood pressure ≥90 mmHg, and unknown); fasting blood glucose (<100, ≥100 mg/dL, and unknown); serum total cholesterol (<200, 200–239, ≥240 mg/dL, and unknown); glomerular filtration rate (<60, 60–89, ≥90 mL/min/1.73 m^2, and unknown); smoking status (never, former, current smoker, and unknown); alcoholic drinks (<1, 1–2, 3–4, ≥5 days per week, and unknown); aerobic physical activity (sufficient, insufficient, and unknown); previous history of cardiovascular disease, and chronic kidney disease; history of medication use for diabetes mellitus, dyslipidemia, and hypertension; and strain of SARS-CoV-2 (original and delta).

Table S19. Stratification analysis for the risk of **acute respiratory complications** following COVID-19 in the propensity score matching cohorts (COVID-19 vs. influenza) of South Korea (main)

	COVID-19 *vs.* influenza (n=223,000)		
		HR (95% CI)	
	Events, *n (%)*	Model 1*	Model 2†
**Sex**			
**Male**			
Comparators (general population or patients with influenza)	23 (0.046)	1.0 (reference)	1.0 (reference)
Patients with COVID-19	51 (0.11)	**27.75 (6.76-114.00)**	**25.32 (6.04-106.13)**
**Female**			
Comparators	22 (0.036)	1.0 (reference)	1.0 (reference)
Patients with COVID-19	64 (0.098)	**10.38 (4.50-23.97)**	**8.23 (3.46-19.58)**
**Age**			
**20–39 y**			
Comparators	18 (0.045)	1.0 (reference)	1.0 (reference)
Patients with COVID-19	15 (0.035)	**6.94 (1.59-30.34)**	**10.78 (2.35-49.52)**
**40–59 years**			
Comparators	12 (0.023)	1.0 (reference)	1.0 (reference)
Patients with COVID-19	43 (0.085)	**46.16 (6.36-335.06)**	**42.17 (5.74-309.82)**
**≥60 y**			
Comparators	15 (0.083)	1.0 (reference)	1.0 (reference)
Patients with COVID-19	57 (0.31)	**11.53 (4.62-28.76)**	**7.80 (3.00-20.28)**
**Region of residence**			
**Rural**			
Comparators	29 (0.059)	1.0 (reference)	1.0 (reference)
Patients with COVID-19	39 (0.079)	**5.57 (2.49-12.46)**	**5.05 (2.18-11.71)**

**Urban**			
Comparators	16 (0.026)	1.0 (reference)	1.0 (reference)
Patients with COVID-19	76 (0.12)	**79.28 (11.03-570.15)**	**65.32 (8.99-474.56)**
**Income level**			
**Low (0th–39th percentile)**			
Comparators	16 (0.035)	1.0 (reference)	1.0 (reference)
Patients with COVID-19	36 (0.074)	**17.62 (4.25-73.15)**	**17.31 (4.07-73.64)**
**Middle (40th –79th percentile)**			
Comparators	21 (0.047)	1.0 (reference)	1.0 (reference)
Patients with COVID-19	55 (0.12)	**9.33 (4.02-21.67)**	**8.26 (3.46-19.72)**
**High (80th–100th percentile)**			
Comparators	8 (0.039)	1.0 (reference)	1.0 (reference)
Patients with COVID-19	24 (0.14)	N/A	N/A
**CCI score**			
**0 score**			
Comparators	37 (0.036)	1.0 (reference)	1.0 (reference)
Patients with COVID-19	61 (0.061)	**8.25 (3.95-17.25)**	**6.79 (3.12-14.75)**
**≥1 scores**			
Comparators	8 (0.10)	1.0 (reference)	1.0 (reference)
Patients with COVID-19	54 (0.46)	N/A	N/A
**BMI**			
**<18.5 kg/m^2**			
Comparators	6 (0.15)	1.0 (reference)	1.0 (reference)
Patients with COVID-19	3 (0.14)	2.84 (0.47-17.04)	2.70 (0.38-19.31)
**18.5-23.0 kg/m^2**			
Comparators	15 (0.038)	1.0 (reference)	1.0 (reference)
Patients with COVID-19	16 (0.070)	**26.54 (3.52-200.29)**	**21.48 (2.83-163.25)**
**23.0-25.0 kg/m^2**			

Comparators	12 (0.049)	1.0 (reference)	1.0 (reference)
Patients with COVID-19	13 (0.091)	**11.02 (2.48-48.95)**	**11.51 (2.57-51.54)**
**≥25.0 kg/m^2**			
Comparators	12 (0.028)	1.0 (reference)	1.0 (reference)
Patients with COVID-19	37 (0.147)	**19.63 (6.05-63.68)**	**16.15 (4.95-52.73)**
**Unknown**			
Comparators	0 (0.00)	1.0 (reference)	1.0 (reference)
Patients with COVID-19	46 (0.10)	N/A	N/A
**Smoking status**			
**Non-smoker**			
Comparators	25 (0.033)	1.0 (reference)	1.0 (reference)
Patients with COVID-19	50 (0.11)	**13.10 (5.61-30.56)**	**11.09 (4.73-25.99)**
**Smoker**			
Comparators	20 (0.055)	1.0 (reference)	1.0 (reference)
Patients with COVID-19	19 (0.10)	**16.91 (3.94-72.67)**	**16.11 (3.73-69.58)**
**Unknown**			
Comparators	0 (0.00)	1.0 (reference)	1.0 (reference)
Patients with COVID-19	46 (0.10)	N/A	N/A
**Alcohol consumption**			
**Non-drinker**			
Comparators	28 (0.041)	1.0 (reference)	1.0 (reference)
Patients with COVID-19	46 (0.12)	**15.83 (6.28-39.88)**	**13.73 (5.43-34.71)**
**Drinker**			
Comparators	17 (0.039)	1.0 (reference)	1.0 (reference)
Patients with COVID-19	23 (0.089)	**11.81 (3.54-39.37)**	**10.20 (3.03-34.35)**
**Unknown**			
Comparators	0 (0.00)	1.0 (reference)	1.0 (reference)
Patients with COVID-19	46 (0.10)	N/A	N/A

**Physical activity**			
**Insufficient physical activity**			
Comparators	23 (0.040)	1.0 (reference)	1.0 (reference)
Patients with COVID-19	36 (0.11)	**18.51 (5.70-60.16)**	**15.87 (4.87-51.79)**
**Sufficient physical activity**			
Comparators	22 (0.041)	1.0 (reference)	1.0 (reference)
Patients with COVID-19	33 (0.11)	**11.29 (4.41-28.94)**	**9.81 (3.80-25.28)**
**Unknown**			
Comparators	0 (0.00)	1.0 (reference)	1.0 (reference)
Patients with COVID-19	46 (0.10)	N/A	N/A

BMI, body mass index; CCI, Charlson comorbidity index; CI, confidence interval; HR, hazard ratio; NA, not available; SARS-CoV-2, severe acute respiratory syndrome coronavirus 2.

Bold indicates that hazard ratio is statistically significant (P<0.05; log-rank test).

***Model 1:** Adjusted for age (20–39, 40–59, and ≥60 years) and sex.

†**Model 2 (main):** Adjusted for age (20–39, 40–59, and ≥60 years); sex, household income (low income, middle income, and high income); region of residence (urban and rural); CCI score (0, 1, and ≥2); obesity (underweight [<18.5 kg/m^2], normal [18.5–22.9 kg/m^2]; overweight [23.0–24.9 kg/m^2], obese [≥25.0 kg/m^2], and unknown); blood pressure (systolic blood pressure <140 mmHg and diastolic blood pressure <90 mmHg, systolic blood pressure ≥140 mmHg or diastolic blood pressure ≥90 mmHg, and unknown); fasting blood glucose (<100, ≥100 mg/dL, and unknown); serum total cholesterol (<200, 200–239, ≥240 mg/dL, and unknown); glomerular filtration rate (<60, 60–89, ≥90 mL/min/1.73 m^2, and unknown); smoking status (never, former, current smoker, and unknown); alcoholic drinks (<1, 1–2, 3–4, ≥5 days per week, and unknown); aerobic physical activity (sufficient, insufficient, and unknown); previous history of cardiovascular disease, and chronic kidney disease; history of medication use for diabetes mellitus, dyslipidemia, and hypertension; and strain of SARS-CoV-2 (original and delta).

Table S20. Stratification analysis for the risk of **post-acute respiratory sequelae** following COVID-19 in the propensity score matching cohorts (COVID-19 vs. general population) of Japan (replication)

	COVID-19 *vs.* general population (n=3,115,606)		
		HR (95% CI)	
	Events, *n (%)*	Model 3*	Model 4ǁ
**Sex**			
**Male**			
Comparators (general population or patients with influenza)	21,197 (1.46)	1.0 (reference)	1.0 (reference)
Patients with COVID-19	24,947 (5.035)	**3.56 (3.49-3.62)**	**3.34 (3.28-3.40)**
**Female**			
Comparators	14,103 (1.62)	1.0 (reference)	1.0 (reference)
Patients with COVID-19	16,127 (5.35)	**3.41 (3.34-3.49)**	**3.29 (3.21-3.36)**
**Age**			
**20–39 y**			
Comparators	12,546 (1.43)	1.0 (reference)	1.0 (reference)
Patients with COVID-19	14,595 (4.83)	**3.47 (3.39-3.56)**	**3.38 (3.30-3.46)**
**40–59 years**			
Comparators	18,329 (1.53)	1.0 (reference)	1.0 (reference)
Patients with COVID-19	20,739 (5.042)	**3.41 (3.35-3.48)**	**3.22 (3.15-3.28)**
**≥60 y**			
Comparators	4425 (1.84)	1.0 (reference)	1.0 (reference)
Patients with COVID-19	5740 (6.89)	**3.93 (3.77-4.08)**	**3.56 (3.42-3.70)**
**CCI score**			
**0 score**			

Comparators	34,452 (1.50)	1.0 (reference)	1.0 (reference)
Patients with COVID-19	36,256 (4.75)	**3.28 (3.23-3.33)**	**3.28 (3.23-3.33)**
**≥1 scores**			
Comparators	848 (3.43)	1.0 (reference)	1.0 (reference)
Patients with COVID-19	4818 (14.58)	**4.15 (3.86-4.47)**	**4.09 (3.80-4.40)**
**BMI**			
**<18.5 kg/m^2**			
Comparators	24,324 (1.43)	1.0 (reference)	1.0 (reference)
Patients with COVID-19	28,166 (4.87)	**3.53 (3.47-3.59)**	**3.36 (3.31-3.42)**
**18.5-23.0 kg/m^2**			
Comparators	8232 (1.70)	1.0 (reference)	1.0 (reference)
Patients with COVID-19	9483 (5.58)	**3.39 (3.29-3.49)**	**3.19 (3.10-3.29)**
**23.0-25.0 kg/m^2**			
Comparators	2681 (2.10)	1.0 (reference)	1.0 (reference)
Patients with COVID-19	3368 (7.13)	**3.45 (3.28-3.63)**	**3.21 (3.05-3.38)**
**≥25.0 kg/m^2**			
Comparators	63 (1.91)	1.0 (reference)	1.0 (reference)
Patients with COVID-19	57 (4.65)	**2.57 (1.80-3.68)**	**2.50 (1.73-3.59)**
**Smoking status**			
**Non-smoker**			
Comparators	34,154 (1.53)	1.0 (reference)	1.0 (reference)
Patients with COVID-19	39,793 (5.18)	**3.50 (3.45-3.55)**	**3.31 (3.26-3.36)**
**Smoker**			
Comparators	1146 (1.32)	1.0 (reference)	1.0 (reference)
Patients with COVID-19	1281 (4.34)	**3.50 (3.23-3.79)**	**3.37 (3.11-3.66)**
**Alcohol consumption**			

**Non-drinker**			
Comparators	18,088 (1.47)	1.0 (reference)	1.0 (reference)
Patients with COVID-19	20,797 (4.88)	**3.44 (3.37-3.51)**	**3.27 (3.20-3.34)**
**Drinker**			
Comparators	14,746 (1.60)	1.0 (reference)	1.0 (reference)
Patients with COVID-19	17,402 (5.58)	**3.58 (3.50-3.66)**	**3.38 (3.31-3.46)**
**Unknown**			
Comparators	2466 (1.45)	1.0 (reference)	1.0 (reference)
Patients with COVID-19	2875 (4.87)	**3.49 (3.31-3.69)**	**3.36 (3.18-3.55)**
**Physical activity**			
**Insufficient physical activity**			
Comparators	7205 (1.45)	1.0 (reference)	1.0 (reference)
Patients with COVID-19	7543 (4.68)	**3.36 (3.25-3.47)**	**3.20 (3.09-3.30)**
**Sufficient physical activity**			
Comparators	25,121 (1.56)	1.0 (reference)	1.0 (reference)
Patients with COVID-19	30,100 (5.35)	**3.52 (3.47-3.58)**	**3.34 (3.28-3.40)**
**Unknown**			
Comparators	2974 (1.38)	1.0 (reference)	1.0 (reference)
Patients with COVID-19	3431 (4.66)	**3.55 (3.38-3.73)**	**3.39 (3.23-3.56)**
**Strain of SARS-CoV-2**			
**Original**			
Comparators	27,023 (2.75)	1.0 (reference)	1.0 (reference)
Patients with COVID-19	29,447 (8.78)	**3.31 (3.26-3.36)**	**3.13 (3.08-3.18)**
**Delta**			
Comparators	8277 (0.62)	1.0 (reference)	1.0 (reference)
Patients with COVID-19	11,627 (2.52)	**4.13 (4.01-4.24)**	**3.94 (3.83-4.05)**

BMI, body mass index; CCI, Charlson comorbidity index; CI, confidence interval; HR, hazard ratio; NA, not available; SARS-CoV-2, severe acute respiratory syndrome coronavirus 2.

Bold indicates that hazard ratio is statistically significant (P<0.05; log-rank test).

***Model 3:** Adjusted for age (20–39, 40–59, and ≥60 years) and sex.

‖ **Model 4 (replication):** Adjusted for age (20–39, 40–59, and ≥60 years); sex; insurance status (insured and dependent); CCI score (0, 1, and ≥ 2); body mass index (underweight [<18.5 kg/m^2], normal [18.5–22.9 kg/m^2], overweight [23.0–25.0 kg/m^2], obese [≥25.0 kg/m^2], and unknown); blood pressure (systolic blood pressure <140 mmHg and diastolic blood pressure <90 mmHg, systolic blood pressure ≥140 mmHg or diastolic blood pressure ≥90 mmHg, and unknown); fasting blood glucose (<100, ≥100 mg/dL, and unknown); serum total cholesterol (<200, 200–239, ≥240 mg/dL, and unknown); glomerular filtration rate (<60, 60–89, ≥90 mL/min/1.73 m^2, and unknown); smoking status (non- and current smoker, and unknown); alcoholic drinks (rarely, sometimes, everyday, and unknown); aerobic physical activity (sufficient, insufficient, and unknown); previous history of cardiovascular disease, and chronic kidney disease; history of medication use for diabetes mellitus, dyslipidemia, and hypertension; and strain of SARS-CoV-2 (original and delta).

Table S21. Stratification analysis for the risk of **post-acute respiratory sequelae** following COVID-19 in the propensity score matching cohorts (COVID-19 vs. influenza) of Japan (replication)

	COVID-19 *vs.* influenza (n=169,924)		
		HR (95% CI)	
	Events, *n (%)*	Model 3*	Model 4ǁ
**Sex**			
**Male**			
Comparators (general population or patients with influenza)	526 (1.00)	1.0 (reference)	1.0 (reference)
Patients with COVID-19	2938 (5.54)	**5.76 (5.25-6.32)**	**5.41 (4.93-5.94)**
**Female**			
Comparators	379 (1.18)	1.0 (reference)	1.0 (reference)
Patients with COVID-19	1819 (5.69)	**5.00 (4.48-5.59)**	**4.83 (4.33-5.40)**
**Age**			
**20–39 y**			
Comparators	373 (1.25)	1.0 (reference)	1.0 (reference)
Patients with COVID-19	1556 (5.19)	**4.29 (3.83-4.80)**	**4.19 (3.75-4.70)**
**40–59 years**			
Comparators	429 (0.92)	1.0 (reference)	1.0 (reference)
Patients with COVID-19	2567 (5.51)	**6.23 (5.63-6.90)**	**5.92 (5.34-6.56)**
**≥60 y**			
Comparators	103 (1.25)	1.0 (reference)	1.0 (reference)
Patients with COVID-19	634 (7.51)	**6.30 (5.11-7.75)**	**5.64 (4.57-6.96)**
**CCI score**			
**0 score**			
Comparators	877 (1.05)	1.0 (reference)	1.0 (reference)
Patients with COVID-19	4196 (5.15)	**5.11 (4.75-5.49)**	**5.11 (4.75-5.50)**

**≥1 scores**			
Comparators	28 (2.43)	1.0 (reference)	1.0 (reference)
Patients with COVID-19	561 (16.01)	**6.61 (4.52-9.66)**	**6.63 (4.53-9.71)**
**BMI**			
**<18.5 kg/m^2**			
Comparators	636 (1.03)	1.0 (reference)	1.0 (reference)
Patients with COVID-19	3290 (5.35)	**5.39 (4.95-5.86)**	**5.16 (4.74-5.62)**
**18.5-23.0 kg/m^2**			
Comparators	195 (1.08)	1.0 (reference)	1.0 (reference)
Patients with COVID-19	1075 (5.88)	**5.67 (4.86-6.60)**	**5.36 (4.59-6.24)**
**23.0-25.0 kg/m^2**			
Comparators	73 (1.47)	1.0 (reference)	1.0 (reference)
Patients with COVID-19	387 (7.58)	**5.26 (4.10-6.76)**	**4.84 (3.76-6.23)**
**≥25.0 kg/m^2**			
Comparators	1 (0.85)	1.0 (reference)	1.0 (reference)
Patients with COVID-19	5 (3.88)	4.87 (0.56-42.31)	3.58 (0.32-40.57)
**Smoking status**			
**Non-smoker**			
Comparators	868 (1.06)	1.0 (reference)	1.0 (reference)
Patients with COVID-19	4604 (5.62)	**5.49 (5.11-5.90)**	**5.20 (4.83-5.59)**
**Smoker**			
Comparators	37 (1.23)	1.0 (reference)	1.0 (reference)
Patients with COVID-19	153 (4.96)	**4.36 (3.04-6.25)**	**4.29 (2.98-6.16)**
**Alcohol consumption**			
**Non-drinker**			
Comparators	443 (0.99)	1.0 (reference)	1.0 (reference)
Patients with COVID-19	2411 (5.30)	**5.58 (5.04-6.17)**	**5.28 (4.77-5.84)**
**Drinker**			
Comparators	397 (1.18)	1.0 (reference)	1.0 (reference)

Patients with COVID-19	2012 (6.03)	**5.28 (4.74-5.88)**	**5.01 (4.50-5.59)**
**Unknown**			
Comparators	65 (1.03)	1.0 (reference)	1.0 (reference)
Patients with COVID-19	334 (5.44)	**5.54 (4.25-7.23)**	**5.53 (4.23-7.22)**
**Physical activity**			
**Insufficient physical activity**			
Comparators	169 (0.97)	1.0 (reference)	1.0 (reference)
Patients with COVID-19	883 (5.10)	**5.50 (4.67-6.49)**	**5.22 (4.43-6.16)**
**Sufficient physical activity**			
Comparators	651 (1.09)	1.0 (reference)	1.0 (reference)
Patients with COVID-19	3477 (5.80)	**5.48 (5.04-5.96)**	**5.20 (4.78-5.65)**
**Unknown**			
Comparators	85 (1.10)	1.0 (reference)	1.0 (reference)
Patients with COVID-19	397 (5.15)	**5.01 (3.96-6.33)**	**4.88 (3.86-6.18)**
**Strain of SARS-CoV-2**			
**Original**			
Comparators	641 (1.80)	1.0 (reference)	1.0 (reference)
Patients with COVID-19	3269 (9.19)	**5.33 (4.89-5.80)**	**5.05 (4.64-5.50)**
**Delta**			
Comparators	264 (0.53)	1.0 (reference)	1.0 (reference)
Patients with COVID-19	1488 (3.01)	**5.73 (5.02-6.53)**	**5.47 (4.79-6.23)**

BMI, body mass index; CCI, Charlson comorbidity index; CI, confidence interval; HR, hazard ratio; NA, not available; SARS-CoV-2, severe acute respiratory syndrome coronavirus 2.

Bold indicates that hazard ratio is statistically significant (P<0.05; log-rank test).

***Model 3:** Adjusted for age (20–39, 40–59, and ≥60 years) and sex.

‖**Model 4 (replication):** Adjusted for age (20–39, 40–59, and ≥60 years); sex; insurance status (insured and dependent); CCI score (0, 1, and ≥ 2); body mass index (underweight [<18.5 kg/m^2], normal [18.5–22.9 kg/m^2], overweight [23.0–25.0 kg/m^2], obese [≥25.0 kg/m^2], and unknown); blood pressure (systolic blood pressure <140 mmHg and diastolic blood pressure <90 mmHg, systolic blood pressure ≥140 mmHg or diastolic blood pressure ≥90 mmHg, and unknown); fasting blood glucose (<100, ≥100 mg/dL, and unknown); serum total cholesterol (<200,

200–239, ≥240 mg/dL, and unknown); glomerular filtration rate (<60, 60–89, ≥90 mL/min/1.73 m^2, and unknown); smoking status (non- and current smoker, and unknown); alcoholic drinks (rarely, sometimes, everyday, and unknown); aerobic physical activity (sufficient, insufficient, and unknown); previous history of cardiovascular disease, and chronic kidney disease; history of medication use for diabetes mellitus, dyslipidemia, and hypertension; and strain of SARS-CoV-2 (original and delta).

Table S22. Stratification analysis for the risk of **acute respiratory complications** following COVID-19 in the propensity score matching cohorts (COVID-19 vs. general population) of Japan (replication)

	COVID-19 *vs.* general population (n=3,115,606)		
		HR (95% CI)	
	Events, *n (%)*	Model 3*	Model 4‖
**Sex**			
**Male**			
Comparators (general population or patients with influenza)	954 (0.066)	1.0 (reference)	1.0 (reference)
Patients with COVID-19	1569 (0.32)	**4.84 (4.46-5.24)**	**4.36 (4.01-4.73)**
**Female**			
Comparators	514 (0.059)	1.0 (reference)	1.0 (reference)
Patients with COVID-19	735 (0.24)	**4.16 (3.72-4.66)**	**3.82 (3.40-4.28)**
**Age**			
**20–39 y**			
Comparators	466 (0.053)	1.0 (reference)	1.0 (reference)
Patients with COVID-19	721 (0.24)	**4.51 (4.01-5.07)**	**4.39 (3.91-4.94)**
**40–59 years**			
Comparators	817 (0.068)	1.0 (reference)	1.0 (reference)
Patients with COVID-19	1176 (0.29)	**4.23 (3.87-4.63)**	**3.77 (3.44-4.13)**
**≥60 y**			
Comparators	185 (0.077)	1.0 (reference)	1.0 (reference)
Patients with COVID-19	407 (0.49)	**6.43 (5.41-7.65)**	**5.33 (4.45-6.37)**
**CCI score**			
**0 score**			
Comparators	1409 (0.061)	1.0 (reference)	1.0 (reference)

Patients with COVID-19	1923 (0.25)	**4.16 (3.88-4.46)**	**4.14 (3.86-4.43)**
**≥1 scores**			
Comparators	59 (0.24)	1.0 (reference)	1.0 (reference)
Patients with COVID-19	381 (1.15)	**4.32 (3.28-5.68)**	**4.40 (3.34-5.79)**
**BMI**			
**<18.5 kg/m^2**			
Comparators	1097 (0.064)	1.0 (reference)	1.0 (reference)
Patients with COVID-19	1636 (0.28)	**4.44 (4.12-4.80)**	**4.05 (3.75-4.38)**
**18.5-23.0 kg/m^2**			
Comparators	288 (0.059)	1.0 (reference)	1.0 (reference)
Patients with COVID-19	508 (0.30)	**5.04 (4.37-5.83)**	**4.51 (3.89-5.22)**
**23.0-25.0 kg/m^2**			
Comparators	82 (0.064)	1.0 (reference)	1.0 (reference)
Patients with COVID-19	154 (0.33)	**4.90 (3.75-6.41)**	**4.37 (3.32-5.74)**
**≥25.0 kg/m^2**			
Comparators	1 (0.030)	1.0 (reference)	1.0 (reference)
Patients with COVID-19	6 (0.49)	**16.98 (2.04-141.11)**	**18.83 (2.12-167.43)**
**Smoking status**			
**Non-smoker**			
Comparators	1396 (0.063)	1.0 (reference)	1.0 (reference)
Patients with COVID-19	2211 (0.29)	**4.63 (4.33-4.95)**	**4.19 (3.91-4.49)**
**Smoker**			
Comparators	72 (0.083)	1.0 (reference)	1.0 (reference)
Patients with COVID-19	93 (0.32)	**4.05 (2.97-5.51)**	**3.84 (2.82-5.24)**
**Alcohol consumption**			
**Non-drinker**			
Comparators	752 (0.061)	1.0 (reference)	1.0 (reference)

Patients with COVID-19	1193 (0.28)	**4.64 (4.24-5.09)**	**4.23 (3.86-4.65)**
**Drinker**			
Comparators	590 (0.064)	1.0 (reference)	1.0 (reference)
Patients with COVID-19	935 (0.30)	**4.65 (4.19-5.15)**	**4.16 (3.75-4.62)**
**Unknown**			
Comparators	126 (0.074)	1.0 (reference)	1.0 (reference)
Patients with COVID-19	176 (0.30)	**4.11 (3.27-5.17)**	**3.91 (3.10-4.92)**
**Physical activity**			
**Insufficient physical activity**			
Comparators	339 (0.068)	1.0 (reference)	1.0 (reference)
Patients with COVID-19	471 (0.29)	**4.36 (3.79-5.02)**	**3.91 (3.39-4.50)**
**Sufficient physical activity**			
Comparators	967 (0.060)	1.0 (reference)	1.0 (reference)
Patients with COVID-19	1610 (0.29)	**4.76 (4.39-5.15)**	**4.30 (3.97-4.66)**
**Unknown**			
Comparators	162 (0.075)	1.0 (reference)	1.0 (reference)
Patients with COVID-19	223 (0.30)	**4.18 (3.42-5.12)**	**3.97 (3.23-4.87)**
**Strain of SARS-CoV-2**			
**Original**			
Comparators	612 (0.062)	1.0 (reference)	1.0 (reference)
Patients with COVID-19	951 (0.28)	**4.58 (4.14-5.07)**	**4.01 (3.61-4.45)**
**Delta**			
Comparators	856 (0.064)	1.0 (reference)	1.0 (reference)
Patients with COVID-19	1353 (0.29)	**4.59 (4.21-5.00)**	**4.28 (3.92-4.66)**

BMI, body mass index; CCI, Charlson comorbidity index; CI, confidence interval; HR, hazard ratio; NA, not available; SARS-CoV-2, severe acute respiratory syndrome coronavirus 2.

Bold indicates that hazard ratio is statistically significant (P<0.05; log-rank test).

*__Model 3:__ Adjusted for age (20–39, 40–59, and ≥60 years) and sex.

‖**Model 4 (replication):** Adjusted for age (20–39, 40–59, and ≥60 years); sex; insurance status (insured and dependent); CCI score (0, 1, and ≥ 2); body mass index (underweight [<18.5 kg/m^2], normal [18.5–22.9 kg/m^2], overweight [23.0–25.0 kg/m^2], obese [≥25.0 kg/m^2], and unknown); blood pressure (systolic blood pressure <140 mmHg and diastolic blood pressure <90 mmHg, systolic blood pressure ≥140 mmHg or diastolic blood pressure ≥90 mmHg, and unknown); fasting blood glucose (<100, ≥100 mg/dL, and unknown); serum total cholesterol (<200, 200–239, ≥240 mg/dL, and unknown); glomerular filtration rate (<60, 60–89, ≥90 mL/min/1.73 m^2, and unknown); smoking status (non- and current smoker, and unknown); alcoholic drinks (rarely, sometimes, everyday, and unknown); aerobic physical activity (sufficient, insufficient, and unknown); previous history of cardiovascular disease, and chronic kidney disease; history of medication use for diabetes mellitus, dyslipidemia, and hypertension; and strain of SARS-CoV-2 (original and delta).

Table S23. Stratification analysis for the risk of **acute respiratory complications** following COVID-19 in the propensity score matching cohorts (COVID-19 vs. influenza) of Japan (replication)

	COVID-19 *vs.* influenza (n=169,924)		
		HR (95% CI)	
	Events, *n (%)*	Model 3*	Model 4‖
**Sex**			
**Male**			
Comparators (general population or patients with influenza)	80 (0.15)	1.0 (reference)	1.0 (reference)
Patients with COVID-19	641 (1.21)	**7.97 (6.32-10.05)**	**6.76 (5.35-8.54)**
**Female**			
Comparators	42 (0.13)	1.0 (reference)	1.0 (reference)
Patients with COVID-19	283 (0.89)	**6.80 (4.92-9.40)**	**6.04 (4.36-8.37)**
**Age**			
**20–39 y**			
Comparators	31 (0.10)	1.0 (reference)	1.0 (reference)
Patients with COVID-19	205 (0.68)	**6.61 (4.53-9.64)**	**6.21 (4.25-9.06)**
**40–59 years**			
Comparators	64 (0.14)	1.0 (reference)	1.0 (reference)
Patients with COVID-19	535 (1.15)	**8.42 (6.50-10.91)**	**7.05 (5.43-9.16)**
**≥60 y**			
Comparators	27 (0.33)	1.0 (reference)	1.0 (reference)
Patients with COVID-19	184 (2.18)	**6.67 (4.45-9.99)**	**5.61 (3.73-8.44)**
**CCI score**			

**0 score**			
Comparators	107 (0.13)	1.0 (reference)	1.0 (reference)
Patients with COVID-19	694 (0.85)	**6.71 (5.47-8.22)**	**6.71 (5.47-8.22)**
**≥1 scores**			
Comparators	15 (1.30)	1.0 (reference)	1.0 (reference)
Patients with COVID-19	230 (6.56)	**4.88 (2.89-8.23)**	**4.82 (2.85-8.16)**
**BMI**			
**<18.5 kg/m^2**			
Comparators	81 (0.13)	1.0 (reference)	1.0 (reference)
Patients with COVID-19	629 (1.02)	**7.84 (6.22-9.88)**	**6.94 (5.50-8.76)**
**18.5-23.0 kg/m^2**			
Comparators	29 (0.16)	1.0 (reference)	1.0 (reference)
Patients with COVID-19	214 (1.17)	**7.24 (4.91-10.67)**	**5.90 (3.99-8.74)**
**23.0-25.0 kg/m^2**			
Comparators	12 (0.24)	1.0 (reference)	1.0 (reference)
Patients with COVID-19	81 (1.59)	**6.52 (3.55-11.95)**	**5.29 (2.86-9.79)**
**≥25.0 kg/m^2**			
Comparators	0 (0.00)	1.0 (reference)	1.0 (reference)
Patients with COVID-19	0 (0.00)	**1.56 (1.54 to 1.59)**	**1.54 (1.51 to 1.57)**
**Smoking status**			
**Non-smoker**			
Comparators	118 (0.14)	1.0 (reference)	1.0 (reference)
Patients with COVID-19	892 (1.09)	**7.57 (6.25-9.17)**	**6.50 (5.36-7.89)**
**Smoker**			
Comparators	4 (0.13)	1.0 (reference)	1.0 (reference)

Patients with COVID-19	32 (1.04)	**8.00 (2.83-22.64)**	**6.43 (2.24-18.45)**
**Alcohol consumption**			
**Non-drinker**			
Comparators	54 (0.12)	1.0 (reference)	1.0 (reference)
Patients with COVID-19	471 (1.04)	**8.64 (6.52-11.45)**	**7.28 (5.48-9.66)**
**Drinker**			
Comparators	60 (0.18)	1.0 (reference)	1.0 (reference)
Patients with COVID-19	398 (1.19)	**6.72 (5.12-8.81)**	**5.87 (4.47-7.72)**
**Unknown**			
Comparators	8 (0.13)	1.0 (reference)	1.0 (reference)
Patients with COVID-19	55 (0.90)	**6.92 (3.30-14.53)**	**6.22 (2.95-13.14)**
**Physical activity**			
**Insufficient physical activity**			
Comparators	24 (0.14)	1.0 (reference)	1.0 (reference)
Patients with COVID-19	184 (1.06)	**7.76 (5.07-11.87)**	**6.78 (4.42-10.41)**
**Sufficient physical activity**			
Comparators	89 (0.15)	1.0 (reference)	1.0 (reference)
Patients with COVID-19	662 (1.10)	**7.42 (5.95-9.26)**	**6.32 (5.06-7.90)**
**Unknown**			
Comparators	9 (0.12)	1.0 (reference)	1.0 (reference)
Patients with COVID-19	78 (1.01)	**8.56 (4.30-17.07)**	**7.55 (3.77-15.11)**
**Strain of SARS-CoV-2**			
**Original**			
Comparators	59 (0.17)	1.0 (reference)	1.0 (reference)
Patients with COVID-19	496 (1.39)	**8.41 (6.42-11.02)**	**6.81 (5.18-8.94)**
**Delta**			

Comparators	63 (0.13)	1.0 (reference)	1.0 (reference)
Patients with COVID-19	428 (0.87)	**6.80 (5.22-8.85)**	**6.21 (4.76-8.10)**

BMI, body mass index; CCI, Charlson comorbidity index; CI, confidence interval; HR, hazard ratio; NA, not available; SARS-CoV-2, severe acute respiratory syndrome coronavirus 2.

Bold indicates that hazard ratio is statistically significant (P<0.05; log-rank test).

***Model 3:** Adjusted for age (20–39, 40–59, and ≥60 years) and sex.

‖**Model 4 (replication):** Adjusted for age (20–39, 40–59, and ≥60 years); sex; insurance status (insured and dependent); CCI score (0, 1, and ≥ 2); body mass index (underweight [<18.5 kg/m^2], normal [18.5–22.9 kg/m^2], overweight [23.0–25.0 kg/m^2], obese [≥25.0 kg/m^2], and unknown); blood pressure (systolic blood pressure <140 mmHg and diastolic blood pressure <90 mmHg, systolic blood pressure ≥140 mmHg or diastolic blood pressure ≥90 mmHg, and unknown); fasting blood glucose (<100, ≥100 mg/dL, and unknown); serum total cholesterol (<200, 200–239, ≥240 mg/dL, and unknown); glomerular filtration rate (<60, 60–89, ≥90 mL/min/1.73 m^2, and unknown); smoking status (non- and current smoker, and unknown); alcoholic drinks (rarely, sometimes, everyday, and unknown); aerobic physical activity (sufficient, insufficient, and unknown); previous history of cardiovascular disease, and chronic kidney disease; history of medication use for diabetes mellitus, dyslipidemia, and hypertension; and strain of SARS-CoV-2 (original and delta).

Table S24. HR (95% CI) for the **post-acute respiratory sequelae** or **acute respiratory complications** subtypes after the PCR test in the propensity score-matched cohorts (COVID-19 vs. general population) in South Korea

	COVID-19 vs. general population (n=492,774)		
		HR (95% CI)	
	Events, *n (%)*	Model 1*	Model 2†
**Post-acute respiratory sequelae**			
Comparators	1745 (0.43)	1.0 (reference)	1.0 (reference)
Patients with COVID-19	1675 (2.02)	**7.95 (7.37-8.58)**	**8.10 (7.45-8.80)**
**Chronic respiratory failure**			
Comparators	9 (0.0022)	1.0 (reference)	1.0 (reference)
Patients with COVID-19	33 (0.039)	**21.16 (9.77-45.81)**	**19.64 (8.66-44.54)**
**Pulmonary hypertension**			
Comparators	5 (0.0012)	1.0 (reference)	1.0 (reference)
Patients with COVID-19	1 (0.0012)	1.68 (0.17-16.10)	0.33 (0.02-4.72)
**Sleep apnea**			
Comparators	118 (0.029)	1.0 (reference)	1.0 (reference)
Patients with COVID-19	78 (0.094)	**5.65 (4.09-7.80)**	**6.47 (4.58-9.14)**
**COPD**			
Comparators	1154 (0.28)	1.0 (reference)	1.0 (reference)
Patients with COVID-19	1047 (1.26)	**7.32 (6.66-8.04)**	**7.37 (6.65-8.17)**
**Emphysema**			
Comparators	70 (0.0017)	1.0 (reference)	1.0 (reference)
Patients with COVID-19	56 (0.068)	**7.18 (4.79-10.77)**	**7.42 (4.81-11.45)**

**Asthma**			
Comparators	435 (0.11)	1.0 (reference)	1.0 (reference)
Patients with COVID-19	443 (0.53)	**8.28 (7.12-9.63)**	**8.46 (7.19-9.96)**
**Pulmonary sarcoidosis**			
Comparators	8 (0.0020)	1.0 (reference)	1.0 (reference)
Patients with COVID-19	2 (0.0024)	2.52 (0.46-13.74)	4.20 (0.76-23.20)
**Interstitial lung disease**			
Comparators	96 (0.023)	1.0 (reference)	1.0 (reference)
Patients with COVID-19	177 (0.21)	**19.32 (14.01-26.65)**	**21.12 (15.15-29.45)**
**Acute respiratory complications**			
Comparators	135 (0.033)	1.0 (reference)	1.0 (reference)
Patients with COVID-19	174 (0.21)	**6.51 (5.20-8.16)**	**5.26 (4.07-6.80)**
**Pneumocystis pneumonia**			
Comparators	0 (0.00)	1.0 (reference)	1.0 (reference)
Patients with COVID-19	0 (0.00)	NA	NA
**Aspergillosis pneumonia**			
Comparators	9 (0.0022)	1.0 (reference)	1.0 (reference)
Patients with COVID-19	10 (0.012)	**5.68 (2.31-13.97)**	**5.54 (2.08-14.74)**
**Pleural empyema**			
Comparators	6 (0.0015)	1.0 (reference)	1.0 (reference)
Patients with COVID-19	3 (0.0036)	2.53 (0.63-10.10)	0.71 (0.13-4.00)
**Lung abscess**			
Comparators	1 (0.00024)	1.0 (reference)	1.0 (reference)
Patients with COVID-19	1 (0.0012)	4.92 (0.31-78.60)	15.36 (0.74-319.84)
**Pneumothorax**			
Comparators	16 (0.0039)	1.0 (reference)	1.0 (reference)

Patients with COVID-19	13 (0.016)	**4.09 (1.97-8.50)**	**3.80 (1.65-8.73)**
**Acute respiratory failure**			
Comparators	48 (0.012)	1.0 (reference)	1.0 (reference)
Patients with COVID-19	102 (0.12)	**10.70 (7.59-15.08)**	**7.78 (5.29-11.46)**
**Pulmonary embolism**			
Comparators	56 (0.014)	1.0 (reference)	1.0 (reference)
Patients with COVID-19	45 (0.054)	**4.07 (2.75-6.02)**	**3.72 (2.38-5.82)**

CCI, Charlson comorbidity index; CI, confidence interval; COPD, chronic obstructive pulmonary disease; HR, hazard ratio; NA, not available; PCR, polymerase chain reaction; SARS-CoV-2, severe acute respiratory syndrome coronavirus 2.

Bold indicates that hazard ratio is statistically significant (P<0.05; log-rank test).

***Model 1:** Adjusted for age (20–39, 40–59, and ≥60 years) and sex.

†**Model 2:** Adjusted for age (20–39, 40–59, and ≥60 years); sex, household income (low income, middle income, and high income); region of residence (urban and rural); CCI score (0, 1, and ≥2); obesity (underweight [<18.5 kg/m^2], normal [18.5–22.9 kg/m^2]; overweight [23.0–24.9 kg/m^2], obese [≥25.0 kg/m^2], and unknown); blood pressure (systolic blood pressure <140 mmHg and diastolic blood pressure <90 mmHg, systolic blood pressure ≥140 mmHg or diastolic blood pressure ≥90 mmHg, and unknown); fasting blood glucose (<100, ≥100 mg/dL, and unknown); serum total cholesterol (<200, 200–239, ≥240 mg/dL, and unknown); glomerular filtration rate (<60, 60–89, ≥90 mL/min/1.73 m^2, and unknown); smoking status (never, former, current smoker, and unknown); alcoholic drinks (<1, 1–2, 3–4, ≥5 days per week, and unknown); aerobic physical activity (sufficient, insufficient, and unknown); previous history of cardiovascular disease, and chronic kidney disease; history of medication use for diabetes mellitus, dyslipidemia, and hypertension; and strain of SARS-CoV-2 (original and delta).

Table S25. Definitions of diseases

**Diseases**	**ICD-10 codes**
Chronic respiratory failure	J96.1 with ≥1 claims
Pulmonary hypertension	I27.0 with ≥1 claims
Sleep apnea	G47.3 with ≥1 claims

Chronic obstructive pulmonary disease	J40, J41, J42 or J44 with ≥1 claims
Emphysema	J43 or J98.2 with ≥1 claims
Asthma	J45 or J46 with ≥1 claims
Pulmonary sarcoidosis	D86 with ≥1 claims
Interstitial lung disease	J84 with ≥1 claims
Pneumocystis pneumonia	B20.6 or B59 with ≥1 claims
Aspergillosis pneumonia	B44.0 or B44.1 with ≥1 claims
Pleural empyema	J86 with ≥1 claims
Lung abscess	J85 with ≥1 claims
Pneumothorax	J93 with ≥1 claims
Acute respiratory failure	J96 with ≥1 claims
Pulmonary embolism	I26 with ≥1 claims
Tympanic membrane perforation	H72 with ≥1 claims

ICD-10, International Classification of Diseases, 10th edition.

Table S26. Statistical analyses and justification

**No.** Statistical analysis (1 to **9)**	Cohort	Justification
**1.** Incident respiratory diseases in COVID-19 versus general population	1. Main cohort after 1:5 propensity score matching<br>2. Replication cohort 1:3 propensity score matching	- Main results<br>- To investigate the association of acute respiratory complications and post-acute respiratory sequelae.<br><br>- **Matching covariates**: age (20–39, 40–59, and ≥60 years); sex; household income (low income, middle income, and high income); region of residence (urban and rural); previous history of cardiovascular disease, chronic kidney disease; and history of medication use for diabetes mellitus, dyslipidemia, and hypertension.<br>- **Model (main cohort)**: adjusting covariates for age (20–39, 40–59, and ≥60 years); sex, household income (low income, middle income, and high income); region of residence (urban and rural); CCI score (0, 1, and ≥2); obesity (underweight [<18.5 kg/m^2], normal [18.5–22.9 kg/m^2]; overweight [23.0–24.9 kg/m^2], obese [≥25.0 kg/m^2], and unknown); blood pressure (systolic blood pressure <140 mmHg and diastolic blood pressure <90 mmHg, systolic blood pressure ≥140 mmHg or diastolic blood pressure ≥90 mmHg, and unknown); fasting blood glucose (<100, ≥100 mg/dL, and unknown); serum total cholesterol (<200, 200–239, ≥240 mg/dL, and unknown); glomerular filtration rate (<60, 60–89, ≥90 mL/min/1.73 m^2, and unknown); smoking status (never, former, current smoker, and unknown); alcoholic drinks (<1, 1–2, 3–4, ≥5 days per week, and unknown); aerobic physical activity (sufficient, insufficient, and unknown); previous history of cardiovascular disease, and chronic kidney disease; history of medication use for diabetes mellitus, dyslipidemia, and hypertension; and strain of SARS-CoV-2 (original and delta).<br>- **Model (replication cohort)**: adjusting covariates for age (20–39, 40–59, and ≥60 years); sex; insurance status (insured and dependent); CCI score (0, 1, and ≥ 2); body mass index (underweight [<18.5 kg/m^2], normal [18.5–22.9 kg/m^2], overweight [23.0–

		25.0 kg/m^2], obese [≥25.0 kg/m^2], and unknown); blood pressure (systolic blood pressure <140 mmHg and diastolic blood pressure <90 mmHg, systolic blood pressure ≥140 mmHg or diastolic blood pressure ≥90 mmHg, and unknown); fasting blood glucose (<100, ≥100 mg/dL, and unknown); serum total cholesterol (<200, 200–239, ≥240 mg/dL, and unknown); glomerular filtration rate (<60, 60–89, ≥90 mL/min/1.73 m^2, and unknown); smoking status (non- and current smoker, and unknown); alcoholic drinks (rarely, sometimes, everyday, and unknown); aerobic physical activity (sufficient, insufficient, and unknown); previous history of cardiovascular disease, and chronic kidney disease; history of medication use for diabetes mellitus, dyslipidemia, and hypertension; and strain of SARS-CoV-2 (original and delta).
**2.** Subgroup analysis of the risk of several respiratory diseases after SARS-CoV-2 infection	1. Main cohort after 1:5 propensity score matching<br>2. Replication cohort 1:3 propensity score matching	- Main results<br>- To investigate the likelihood of respiratory diseases (post-acute respiratory sequelae and acute respiratory complications) after SARS-CoV-2 infection, compared with general population.<br>- **Post-acute respiratory sequelae**: chronic respiratory failure, pulmonary hypertension, sleep apnea, chronic obstructive pulmonary disease (COPD), emphysema, asthma, pulmonary sarcoidosis, and interstitial lung disease.<br>- **Acute respiratory complications**: pneumocystis pneumonia, aspergillosis pneumonia, pleural empyema, lung abscess, pneumothorax, acute respiratory failure, and pulmonary embolism.
**3.** Subgroup analysis of the risk of incident respiratory diseases after SARS-CoV-2 according to the SARS-CoV-2 vaccinations, severity of COVID-19, and strain type	Main cohort after 1:5 propensity score matching	- Main results<br>- To investigate the association of subsequent all-cause respiratory diseases following COVID-19, stratified by vaccination dose (once and twice or more), and type of vaccinations (vector, mRNA, and mix), severity of COVID-19 (mild and moderate-to-severe), and strain of SARS-CoV-2 (original and delta).
**4.** Attenuation time effect of post-acute respiratory sequelae development after COVID-19	1. Main cohort after 1:5 propensity score matching<br>2. Replication cohort 1:3 propensity score matching	- Main results<br>- To investigate the time attenuation effect of post-acute respiratory sequelae development after SARS-CoV-2 infection (<3, 3 to 6, and ≥6 months).

**5.** Incident respiratory diseases in COVID-19 versus contemporary control (influenza)	1. Main cohort after 1:1 propensity score matching<br>2. Replication cohort 1:1 propensity score matching	- Contemporary control results, consistent with the methodology employed in statistical analysis no. 1, 2, and 4.
**6.** Negative control analysis of the risk of incident tympanic membrane perforation disease.	1. Main cohort after 1:5 propensity score matching<br>2. Replication cohort 1:3 propensity score matching	- To verify the validity of our findings and identify potential misclassification bias, we conducted a negative control analysis of **tympanic membrane perforation disease** following the COVID-19 diagnosis.
**7.** Incident respiratory diseases in positive for COVID-19 versus negative for COVID-19 after the PCR test	Positive for COVID-19 versus negative for COVID-19 after the PCR test after 1:5 propensity score matching in main cohort	- To conduct more sophisticated analysis of the association between SARS-CoV-2 infection and respiratory disease<br>- The cohort was based on the claim record of individuals who tested positive for SARS-CoV-2 within two weeks following an RT-PCR test and those who did not.
**8.** Subgroup analysis of the risk of respiratory diseases after SARS-CoV-2 infection in overlap-weighted cohort	1. Main cohort after overlap-weighted cohort<br>2. Replication cohort after overlap-weighted cohort	- To assess the risks and prevalence of respiratory conditions following COVID-19, the average treatment effect in the overlap (ATO) method was used, with overlap-weighted hazard ratios calculated for precision.<br><br>- **Matching covariates**: age (20–39, 40–59, and ≥60 years); sex; household income (low income, middle income, and high income); region of residence (urban and rural); previous history of cardiovascular disease, chronic kidney disease; and history of medication use for diabetes mellitus, dyslipidemia, and hypertension.<br>- **Model**: adjusting covariates for age (20–39, 40–59, and ≥60 years); sex, household income (low income, middle income, and high income); region of residence (urban and rural); CCI score (0, 1, and ≥2); obesity (underweight [<18.5 kg/m^2], normal [18.5–22.9 kg/m^2]; overweight [23.0–24.9 kg/m^2], obese [≥25.0 kg/m^2], and unknown); blood pressure (systolic blood pressure <140 mmHg and diastolic blood pressure <90 mmHg, systolic blood pressure ≥140 mmHg or diastolic blood pressure ≥90 mmHg, and unknown); fasting blood glucose (<100, ≥100 mg/dL, and unknown); serum total cholesterol (<200, 200–239, ≥240 mg/dL, and unknown); glomerular filtration rate (<60, 60–89, ≥90 mL/min/1.73 m^2, and unknown); smoking status (never, former, current smoker, and unknown); alcoholic drinks (<1, 1–2, 3–4, ≥5 days per week, and unknown); aerobic physical activity (sufficient, insufficient, and unknown); previous

		history of cardiovascular disease, and chronic kidney disease; history of medication use for diabetes mellitus, dyslipidemia, and hypertension; and strain of SARS-CoV-2 (original and delta).
**9**. Stratification analysis of the risk of respiratory diseases and its subtypes development after COVID-19	1. Main cohort after 1:5 propensity score matching<br>2. Replication cohort 1:3 propensity score matching<br>3. Main cohort after 1:1 propensity score matching<br>4. Replication cohort 1:1 propensity score matching	- To investigate unexpected mediated effects by sex, age, region of residence, household income, body mass index, Charlson comorbidity index, smoking status, alcohol use, aerobic physical activity, and strain of SARS-CoV-2.

CCI, Charlson comorbidity index; PCR, polymerase chain reaction; SARS-CoV-2, severe acute respiratory syndrome coronavirus 2.

Table S27. HR (95% CI) for the **post-acute respiratory sequelae** in **negative control analysis** using non-COVID-19 disease (tympanic membrane perforation) in the propensity score-matched main cohort (South Korea) and replication cohort (Japan)

**Cohort**	**South Korea**			**Japan**		
		HR (95% CI)			HR (95% CI)	
	Events, *n (%)*	Model 1*	Model 2†	Events, *n (%)*	Model 3*	Model 4ǁ
**Tympanic membrane perforation**						
Comparators	77 (0.0040)	1.0 (reference)	1.0 (reference)	654 (0.028)	1.0 (reference)	1.0 (reference)
Patients with COVID-19	14 (0.0036)	0.91 (0.51-1.60)	0.72 (0.33-1.56)	294 (0.038)	1.08 (0.69-1.73)	0.94 (0.52-1.80)

CCI, Charlson comorbidity index; CI, confidence interval; HR, hazard ratio.

***Model 1 and 3:** Adjusted for age (20–39, 40–59, and ≥60 years) and sex.

†**Model 2:** Adjusted for age (20–39, 40–59, and ≥60 years); sex, household income (low income, middle income, and high income); region of residence (urban and rural); CCI score (0, 1, and ≥2); obesity (underweight [<18.5 kg/m^2], normal [18.5–22.9 kg/m^2]; overweight [23.0–24.9 kg/m^2], obese [≥25.0 kg/m^2], and unknown); blood pressure (systolic blood pressure <140 mmHg and diastolic blood pressure <90 mmHg, systolic blood pressure ≥140 mmHg or diastolic blood pressure ≥90 mmHg, and unknown); fasting blood glucose (<100, ≥100 mg/dL, and unknown); serum total cholesterol (<200, 200–239, ≥240 mg/dL, and unknown); glomerular filtration rate (<60, 60–89, ≥90 mL/min/1.73 m^2, and unknown); smoking status (never, former, current smoker, and unknown); alcoholic drinks (<1, 1–2, 3–4, ≥5 days per week, and unknown); aerobic physical activity (sufficient, insufficient, and unknown); previous history of cardiovascular disease, and chronic kidney disease; history of medication use for diabetes mellitus, dyslipidemia, and hypertension; and strain of SARS-CoV-2 (original and delta).

ǁ**Model 4:** Adjusted for age (20–39, 40–59, and ≥60 years); sex; insurance status (insured and dependent); CCI score (0, 1, and ≥ 2); body mass index (underweight [<18.5 kg/m^2], normal [18.5–22.9 kg/m^2], overweight [23.0–25.0 kg/m^2], obese [≥25.0 kg/m^2], and unknown); blood pressure (systolic blood pressure <140 mmHg and diastolic blood pressure <90 mmHg, systolic blood pressure ≥140 mmHg or diastolic blood pressure ≥90 mmHg, and unknown); fasting blood glucose (<100, ≥100 mg/dL, and unknown); serum total cholesterol (<200, 200–239, ≥240 mg/dL, and unknown); glomerular filtration rate (<60, 60–89, ≥90 mL/min/1.73 m^2, and unknown); smoking status (non- and current smoker, and unknown); alcoholic drinks (rarely, sometimes, everyday, and unknown); aerobic physical activity (sufficient, insufficient, and unknown); previous history of cardiovascular disease, and chronic kidney disease; history of medication use for diabetes mellitus, dyslipidemia, and

hypertension; and strain of SARS-CoV-2 (original and delta).

Supplementary Material

Description of JMDC cohort

Data source and study design

Japan has health insurance provided by the universal insurance system.[1] The JMDC has agreements with over 60 insurance providers and includes health insurance claims records of insured individuals, mostly employees of relatively large companies in Japan.[2] JMDC has generated a database, using data collected from medical institutions in Japan, consisting of patient-level data (unique identifier, family identifiers, relationship to the insured individual, age, sex) and claims for inpatient and outpatient treatment (disease class according to International Classification of Diseases [ICD]-10 code, prescribed drugs based on Anatomical Therapeutic Chemical class, and drug dosage form), diagnosis or therapeutic procedure, institutional information (hospital character), and health checkup (i.e., body mass index, blood pressure, clinical laboratory test, medication status, and self-administered questionnaire such as smoking status, alcohol consumption, and physical activity).[3] For more information, please see this webpage (https://www.jmdc.co.jp/en/jmdc-claims-database/). Similar to the South Korea cohort, we collected a total of 4,909,861 subjects aged ≥20 years who had information on medical examinations in the JMDC data from January 1, 2018, to December 31, 2021. We excluded participants with the following criteria: (1) insufficient demographic information and those who died before (excluded n=916,070); and (2) previous history of chronic respiratory disease in the pre-observation period (excluded n=386,937).

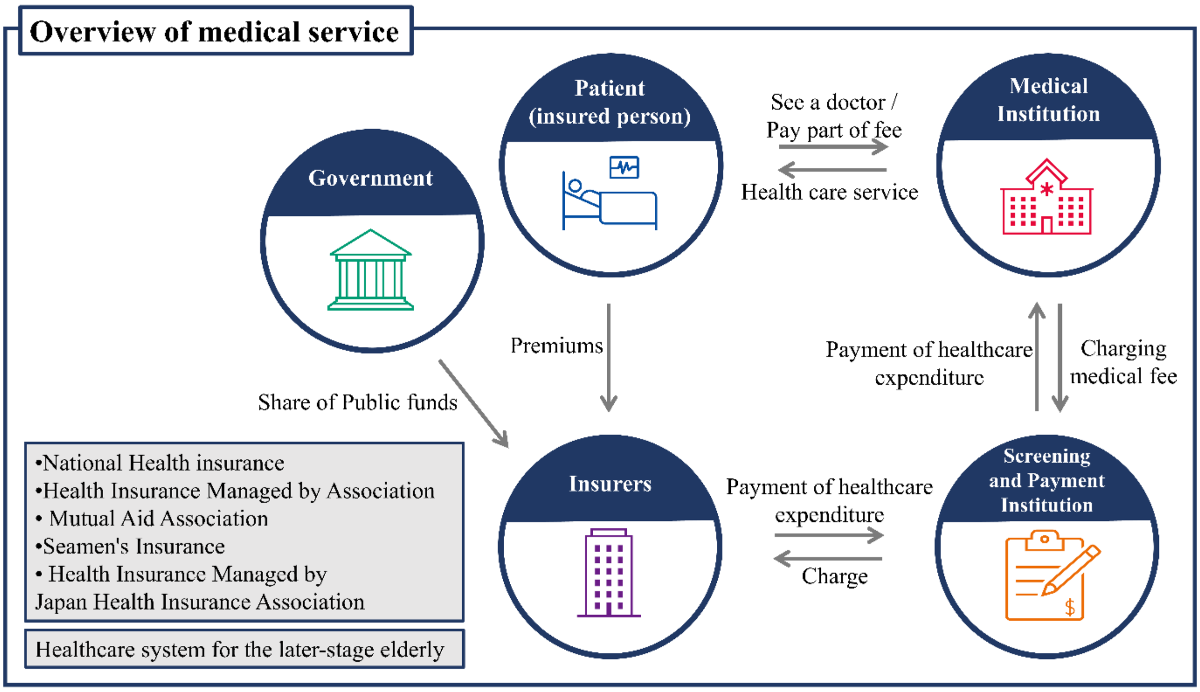
Overview of medical service
Patient (insured person)
See a doctor / Pay part of fee
Health care service
Medical Institution
Government
Premiums
Share of Public funds
Payment of healthcare expenditure
Charging medical fee
•National Health insurance
•Health Insurance Managed by Association
• Mutual Aid Association
•Seamen's Insurance
• Health Insurance Managed by Japan Health Insurance Association
Healthcare system for the later-stage elderly
Insurers
Payment of healthcare expenditure
Charge
Screening and Payment Institution

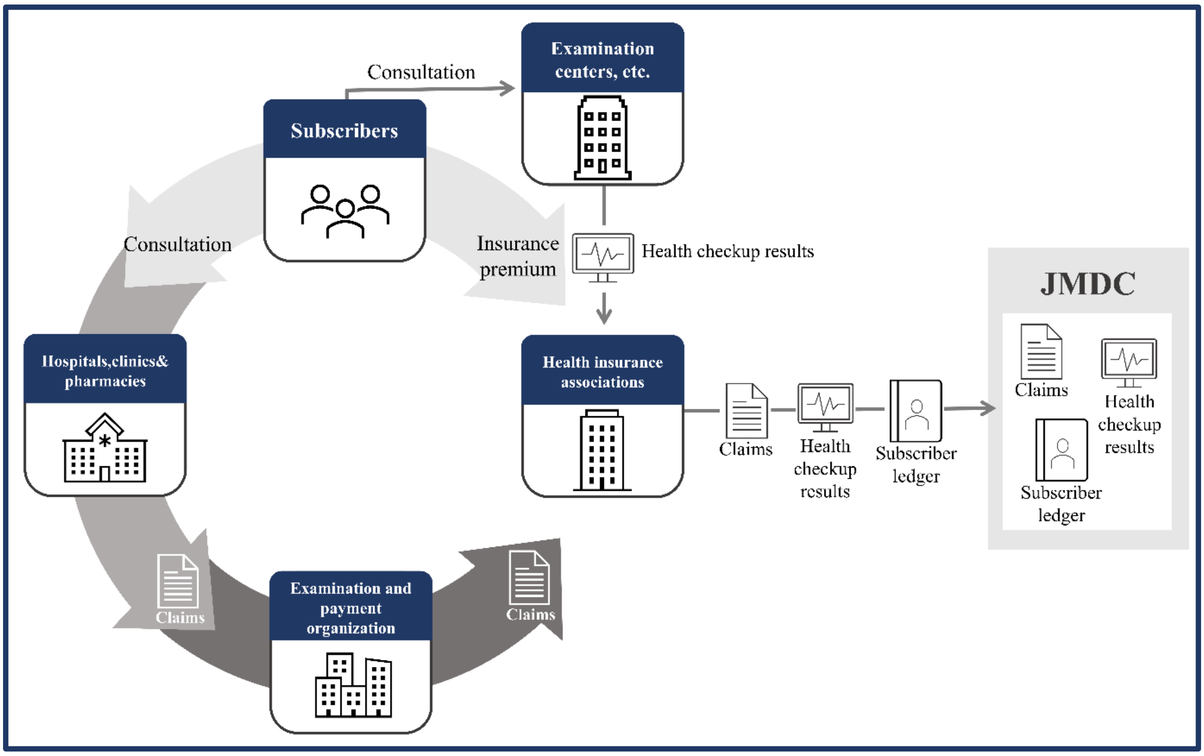
Examination centers, etc.
Consultation
Subscribers
Consultation
Insurance premium
Health checkup results
JMDC
Claims
Health checkup results
Subscriber ledger
Hospitals,clinics& pharmacies
Health insurance associations
Claims
Health checkup results
Subscriber ledger
Claims
Examination and payment organization
Claims

Covariates

Participant demographic data was sourced from the JMDC database, which included age (20–39, 40–59, and ≥60 years), sex, insurance status (insured and dependent), CCI score (0, 1, and ≥ 2); body mass index (underweight [<18.5 kg/m^2], normal [18.5–22.9 kg/m^2], overweight [23.0–25.0 kg/m^2], obese [≥25.0 kg/m^2], and unknown), blood pressure (systolic blood pressure <140 mmHg and diastolic blood pressure <90 mmHg, systolic blood pressure ≥140 mmHg or diastolic blood pressure ≥90 mmHg, and unknown), fasting blood glucose (<100, ≥100 mg/dL, and unknown), serum total cholesterol (<200, 200–239, ≥240 mg/dL, and unknown), glomerular filtration rate (<60, 60–89, ≥90 mL/min/1.73 m^2, and unknown), smoking status (non- and current smoker, and unknown), alcoholic drinks (rarely, sometimes, everyday, and unknown), aerobic physical activity (sufficient, insufficient, and unknown), previous history of cardiovascular disease and chronic kidney disease, history of medication use for diabetes mellitus, dyslipidemia, and hypertension, and strain of SARS-CoV-2 (original and delta).

Reference

1 Reich, M. R., Ikegami, N., Shibuya, K. & Takemi, K. 50 years of pursuing a healthy society in Japan. *Lancet* **378**, 1051-1053 (2011). https://doi.org/10.1016/s0140-6736(11)60274-2

2 Setogawa, N., Ohbe, H., Isogai, T., Matsui, H. & Yasunaga, H. Characteristics and short-term outcomes of outpatient and inpatient cardiac catheterizations: A descriptive study using a nationwide claim database in Japan. *J Cardiol* **82**, 201-206 (2023). https://doi.org/10.1016/j.jjcc.2023.05.010

3 Kaneko, H. *et al.* Medication-Naïve Blood Pressure and Incident Cancers: Analysis of 2 Nationwide Population-Based Databases. *Am J Hypertens* **35**, 731-739 (2022). https://doi.org/10.1093/ajh/hpac054
