# Supplementary material for: Acute and post-acute respiratory complications of SARS-CoV-2 infection: population-based cohort study in South Korea and Japan
Source: Nat Commun. 2024 May 27;15:4499. doi: 10.1038/s41467-024-48825-w (PMC11130304; doi:10.1038/s41467-024-48825-w)
Supplement: Supplementary file 3 — Reporting Summary [file 41467_2024_48825_MOESM3_ESM.pdf]

nature portfolio

Corresponding author(s): Hayeon Lee, Jiseung Kang, and Dong Keon Yon

Last updated by author(s): May 6, 2024

# Reporting Summary

Nature Portfolio wishes to improve the reproducibility of the work that we publish. This form provides structure for consistency and transparency in reporting. For further information on Nature Portfolio policies, see our Editorial Policies and the Editorial Policy Checklist.

## Statistics

For all statistical analyses, confirm that the following items are present in the figure legend, table legend, main text, or Methods section.

| n/a | Confirmed | |
|---|---|---|
| ☐ | ☒ | The exact sample size (*n*) for each experimental group/condition, given as a discrete number and unit of measurement |
| ☐ | ☒ | A statement on whether measurements were taken from distinct samples or whether the same sample was measured repeatedly |
| ☐ | ☒ | The statistical test(s) used AND whether they are one- or two-sided<br>*Only common tests should be described solely by name; describe more complex techniques in the Methods section.* |
| ☐ | ☒ | A description of all covariates tested |
| ☐ | ☒ | A description of any assumptions or corrections, such as tests of normality and adjustment for multiple comparisons |
| ☐ | ☒ | A full description of the statistical parameters including central tendency (e.g. means) or other basic estimates (e.g. regression coefficient) AND variation (e.g. standard deviation) or associated estimates of uncertainty (e.g. confidence intervals) |
| ☐ | ☒ | For null hypothesis testing, the test statistic (e.g. *F*, *t*, *r*) with confidence intervals, effect sizes, degrees of freedom and *P* value noted<br>*Give P values as exact values whenever suitable.* |
| ☒ | ☐ | For Bayesian analysis, information on the choice of priors and Markov chain Monte Carlo settings |
| ☒ | ☐ | For hierarchical and complex designs, identification of the appropriate level for tests and full reporting of outcomes |
| ☒ | ☐ | Estimates of effect sizes (e.g. Cohen's *d*, Pearson's *r*), indicating how they were calculated |

*Our web collection on statistics for biologists contains articles on many of the points above.*

## Software and code

Policy information about availability of computer code

| | |
|---|---|
| Data collection | Data collection were performed using SAS (version 9.4; SAS Institute Inc., Cary, NC, USA). |
| Data analysis | Statistical analyses were performed using SAS (version 9.4; SAS Institute Inc., Cary, NC, USA) for big-data analysis. Within each cohort, a nearest-neighbor algorithm was used to perform exposure-driven propensity score matching between the two groups based on exposure status. This matching involved a random selection without replacement, constrained by specified caliper widths of 0.001 standard deviations. Hazard ratios (HRs) with 95% confidence intervals (CIs) using cox proportional hazard regression models were used for estimation, and were executed using SAS. |

For manuscripts utilizing custom algorithms or software that are central to the research but not yet described in published literature, software must be made available to editors and reviewers. We strongly encourage code deposition in a community repository (e.g. GitHub). See the Nature Portfolio guidelines for submitting code & software for further information.

## Data

Policy information about availability of data

All manuscripts must include a data availability statement. This statement should provide the following information, where applicable:

- Accession codes, unique identifiers, or web links for publicly available datasets
- A description of any restrictions on data availability
- For clinical datasets or third party data, please ensure that the statement adheres to our policy

The datasets analysed during the current study are available in the National Health Insurance Service, South Korea (https://nhiss.nhis.or.kr/bd/ab/bdaba000eng.do) and the JMDC, Japan (https://www.jmdc.co.jp/en/jmdc-claims-database/). This protects the confidentiality of the data and ensures that Information Governance is robust. Applications to access health data in South Korea are submitted to the National Health Insurance Service, South Korea. Information can be found at https://nhiss.nhis.or.kr/bd/ab/bdaba000eng.do. Applications to access health data in Japan are submitted to the JMDC, Japan. Information can be found at https://www.jmdc.co.jp/en/jmdc-claims-database/.

## Research involving human participants, their data, or biological material

Policy information about studies with human participants or human data. See also policy information about sex, gender (identity/presentation), and sexual orientation and race, ethnicity and racism.

Reporting on sex and gender

We used the population-based nationwide National Health Information Database (Korea Disease Control and Prevention Agency-COIVD-19-National Health Insurance Service cohort [K-COV-N cohort]) in South Korea and Japanese claim-based cohort (JMDC cohort) in Japan. Thus, this is determined by the "sex" of a participant reported to the government system.

Reporting on race, ethnicity, or other socially relevant groupings

For the study, the claims based data of South Korea and Japan were used to comprehensively investigate the association of the study outcome.

We utilized socially relevant variables officially reported in each database, which were categorized based on prior research that considered biases in healthcare access and social environments.
- Main cohort (South Korea): household income percentiles (low [0–39], middle [40–79], high [80–100]); region of residence (urban and rural)
- Replication cohort (Japan): insurance status (insured and dependent)

Following variables were used to adjust confounding factors in the study:
- Main cohort (South Korea): age (20–39, 40–59, and ≥60 years); sex, household income percentiles (low [0–39], middle [40–79], high [80–100]); region of residence (urban and rural); Charlson comorbidity index (CCI) score (0, 1, and ≥2); obesity (underweight [<18.5 kg/m2], normal [18.5–22.9 kg/m2]; overweight [23.0–24.9 kg/m2], obese [≥25.0 kg/m2], and unknown); blood pressure (systolic blood pressure <140 mmHg and diastolic blood pressure <90 mmHg, systolic blood pressure ≥140 mmHg or diastolic blood pressure ≥90 mmHg, and unknown); fasting blood glucose (<100, ≥100 mg/dL, and unknown); serum total cholesterol (<200, 200–239, ≥240 mg/dL, and unknown); glomerular filtration rate (<60, 60–89, ≥90 mL/min/1.73 m2, and unknown); smoking status (never, former, current smoker, and unknown); alcoholic drinks (<1, 1–2, 3–4, ≥5 days per week, and unknown); aerobic physical activity (sufficient, insufficient, and unknown); previous history of cardiovascular disease, and chronic kidney disease; history of medication use for diabetes mellitus, dyslipidemia, and hypertension; and strain of SARS-CoV-2 (original and delta).
- Replication cohort (Japan): age (20–39, 40–59, and ≥60 years); sex; insurance status (insured and dependent); CCI score (0, 1, and ≥ 2); body mass index (underweight [<18.5 kg/m2], normal [18.5–22.9 kg/m2], overweight [23.0–25.0 kg/m2], obese [≥25.0 kg/m2], and unknown); blood pressure (systolic blood pressure <140 mmHg and diastolic blood pressure <90 mmHg, systolic blood pressure ≥140 mmHg or diastolic blood pressure ≥90 mmHg, and unknown); fasting blood glucose (<100, ≥100 mg/dL, and unknown); serum total cholesterol (<200, 200–239, ≥240 mg/dL, and unknown); glomerular filtration rate (<60, 60–89, ≥90 mL/min/1.73 m2, and unknown); smoking status (non- and current smoker, and unknown); alcoholic drinks (rarely, sometimes, everyday, and unknown); aerobic physical activity (sufficient, insufficient, and unknown); previous history of cardiovascular disease, and chronic kidney disease; history of medication use for diabetes mellitus, dyslipidemia, and hypertension; and strain of SARS-CoV-2 (original and delta).

Population characteristics

The dataset was linked and consisted of data on first general health examination results, death records, health insurance data including insurance eligibility data, personal sociodemographic data, inpatient and outpatient healthcare records, and medication records.

- Main cohort (South Korea): age (20–39, 40–59, and ≥60 years); sex, household income (low income, middle income, and high income); region of residence (urban and rural); Charlson comorbidity index (CCI) score (0, 1, and ≥2); obesity (underweight [<18.5 kg/m2], normal [18.5–22.9 kg/m2]; overweight [23.0–24.9 kg/m2], obese [≥25.0 kg/m2], and unknown); blood pressure (systolic blood pressure <140 mmHg and diastolic blood pressure <90 mmHg, systolic blood pressure ≥140 mmHg or diastolic blood pressure ≥90 mmHg, and unknown); fasting blood glucose (<100, ≥100 mg/dL, and unknown); serum total cholesterol (<200, 200–239, ≥240 mg/dL, and unknown); glomerular filtration rate (<60, 60–89, ≥90 mL/min/1.73 m2, and unknown); smoking status (never, former, current smoker, and unknown); alcoholic drinks (<1, 1–2, 3–4, ≥5 days per week, and unknown); aerobic physical activity (sufficient, insufficient, and unknown); previous history of cardiovascular disease, and chronic kidney disease; history of medication use for diabetes mellitus, dyslipidemia, and hypertension; and strain of SARS-CoV-2 (original and delta).
- Replication cohort (Japan): age (20–39, 40–59, and ≥60 years); sex; insurance status (insured and dependent); CCI score (0, 1, and ≥ 2); body mass index (underweight [<18.5 kg/m2], normal [18.5–22.9 kg/m2], overweight [23.0–25.0 kg/m2], obese [≥25.0 kg/m2], and unknown); blood pressure (systolic blood pressure <140 mmHg and diastolic blood pressure <90 mmHg, systolic blood pressure ≥140 mmHg or diastolic blood pressure ≥90 mmHg, and unknown); fasting blood glucose (<100, ≥100

| | |
|---|---|
| | mg/dL, and unknown); serum total cholesterol (<200, 200–239, ≥240 mg/dL, and unknown); glomerular filtration rate (<60, 60–89, ≥90 mL/min/1.73 m2, and unknown); smoking status (non- and current smoker, and unknown); alcoholic drinks (rarely, sometimes, everyday, and unknown); aerobic physical activity (sufficient, insufficient, and unknown); previous history of cardiovascular disease, and chronic kidney disease; history of medication use for diabetes mellitus, dyslipidemia, and hypertension; and strain of SARS-CoV-2 (original and delta). |
| Recruitment | The study was a bi-national population-based cohort study that included all adults (aged ≥ 20 years) reported to each agency during COVID-19, each receiving a medical examination. We used the South Korean population-based cohort (K-CoV-N; N=10,027,506) as a main cohort and the Japanese claims-based cohort (JMDC; N=4,909,861) as replication cohort. We assessed the risk of incident various respiratory disease immediately after SARS-CoV-2 infection compared with contemporary controls who were not infected. The pre-observation period to determine the previous diagnostic history was from 2018 to 2019, and the follow-up period was from 2020 to 2021. Individuals with missing socioeconomic status data, those who died, or those with a history of allergy disease during the pre-observation period were excluded from the analysis in K-CoV-N (excluded, n=4,423,753) and JMDC (excluded, n=1,122,179). |
| Ethics oversight | This study received approvals from the Korea Disease Control and Prevention Agency (KDCA), National Health Insurance Service (NHIS; KDCA-NHIS-2022-1-632), JMDC (PHP-00002201-04), and the Institutional Review Board of Kyung Hee University (KHSIRB-23-241). Under the terms of the approval, patient consent was not required for the use of routine health records for our study. |

Note that full information on the approval of the study protocol must also be provided in the manuscript.

# Field-specific reporting

Please select the one below that is the best fit for your research. If you are not sure, read the appropriate sections before making your selection.

☒ Life sciences ☐ Behavioural & social sciences ☐ Ecological, evolutionary & environmental sciences

For a reference copy of the document with all sections, see nature.com/documents/nr-reporting-summary-flat.pdf

# Life sciences study design

All studies must disclose on these points even when the disclosure is negative.

| | |
|---|---|
| Sample size | This study utilized large-scale, population-based, bi-national cohorts, including a South Korean claims-based nationwide cohort (K-CoV-N; N=10,027,506) as a main cohort and a Japanese claims-based cohort (JMDC; N=4,909,861) as a replication cohort. They are collected over the observation period from January 1, 2018, to December 31, 2021, among individuals aged 20 and above, with each receiving a medical examination. |
| Data exclusions | Individuals with missing socioeconomic status data, those who died, or those with a history of allergy disease during the pre-observation period were excluded from the analysis in K-CoV-N (excluded, n=4,423,753) and JMDC (excluded, n=1,122,179). |
| Replication | Similar findings were reported in the replication cohorts.<br>A Cox proportional hazards regression model with estimates of HRs and 95% CIs was used to explore incident acute respiratory complications and long-term respiratory sequelae associated with long COVID. Models were adjusted for following variables:<br><br>- Model (main cohort; South Korea): Adjusted for age (20–39, 40–59, and ≥60 years); sex, household income (low income, middle income, and high income); region of residence (urban and rural); Charlson comorbidity index (CCI) score (0, 1, and ≥2); obesity (underweight [<18.5 kg/m2], normal [18.5–22.9 kg/m2]; overweight [23.0–24.9 kg/m2], obese [≥25.0 kg/m2], and unknown); blood pressure (systolic blood pressure <140 mmHg and diastolic blood pressure <90 mmHg, systolic blood pressure ≥140 mmHg or diastolic blood pressure ≥90 mmHg, and unknown); fasting blood glucose (<100, ≥100 mg/dL, and unknown); serum total cholesterol (<200, 200–239, ≥240 mg/dL, and unknown); glomerular filtration rate (<60, 60–89, ≥90 mL/min/1.73 m2, and unknown); smoking status (never, former, current smoker, and unknown); alcoholic drinks (<1, 1–2, 3–4, ≥5 days per week, and unknown); aerobic physical activity (sufficient, insufficient, and unknown); previous history of cardiovascular disease, and chronic kidney disease; history of medication use for diabetes mellitus, dyslipidemia, and hypertension; and strain of SARS-CoV-2 (original and delta).<br>- Model (replication cohort; Japan): Adjusted for age (20–39, 40–59, and ≥60 years); sex; insurance status (insured and dependent); CCI score (0, 1, and ≥ 2); body mass index (underweight [<18.5 kg/m2], normal [18.5–22.9 kg/m2], overweight [23.0–25.0 kg/m2], obese [≥25.0 kg/m2], and unknown); blood pressure (systolic blood pressure <140 mmHg and diastolic blood pressure <90 mmHg, systolic blood pressure ≥140 mmHg or diastolic blood pressure ≥90 mmHg, and unknown); fasting blood glucose (<100, ≥100 mg/dL, and unknown); serum total cholesterol (<200, 200–239, ≥240 mg/dL, and unknown); glomerular filtration rate (<60, 60–89, ≥90 mL/min/1.73 m2, and unknown); smoking status (non- and current smoker, and unknown); alcoholic drinks (rarely, sometimes, everyday, and unknown); aerobic physical activity (sufficient, insufficient, and unknown); previous history of cardiovascular disease, and chronic kidney disease; history of medication use for diabetes mellitus, dyslipidemia, and hypertension; and strain of SARS-CoV-2 (original and delta).<br><br>We performed multi-to-one exposure-driven propensity score matching in the nationwide cohorts of South Korea and Japan. Propensity score was derived using a binary logistic regression model adjusted for variables such as age, sex, household income, region, previous history of cardiovascular and chronic kidney disease, and history of medication use for diabetes mellitus, dyslipidemia, and hypertension. |
| Randomization | Not applicable; individuals were recruited in an observational study with convenience samples being collected. |
| Blinding | Blinding was not relevant, since this is an observational study, where participants were invited based on test status (case or control). |

# Reporting for specific materials, systems and methods

We require information from authors about some types of materials, experimental systems and methods used in many studies. Here, indicate whether each material, system or method listed is relevant to your study. If you are not sure if a list item applies to your research, read the appropriate section before selecting a response.

## Materials & experimental systems

| n/a | Involved in the study |
|---|---|
| ☒ | ☐ Antibodies |
| ☒ | ☐ Eukaryotic cell lines |
| ☒ | ☐ Palaeontology and archaeology |
| ☒ | ☐ Animals and other organisms |
| ☒ | ☐ Clinical data |
| ☒ | ☐ Dual use research of concern |
| ☒ | ☐ Plants |

## Methods

| n/a | Involved in the study |
|---|---|
| ☒ | ☐ ChIP-seq |
| ☒ | ☐ Flow cytometry |
| ☒ | ☐ MRI-based neuroimaging |

## Plants

| | |
|---|---|
| Seed stocks | Not applicable. |
| Novel plant genotypes | Not applicable. |
| Authentication | Not applicable. |
